# The development of national growth charts for Jordanian children aged 0–2 years

**DOI:** 10.3389/fped.2025.1547581

**Published:** 2025-08-12

**Authors:** Walid Al-Qerem, Lina Bataineh, Anan Jarab, Judith Eberhardt, Fawaz Alasmari, Alaa Hammad

**Affiliations:** ^1^ Department of Pharmacy, Faculty of Pharmacy, Al-Zaytoonah University of Jordan, Amman, Jordan; ^2^ Department of Clinical Pharmacy, Faculty of Pharmacy, Jordan University of Science and Technology, Irbid, Jordan; ^3^ Department of Psychology, School of Social Sciences, Humanities and Law, Teesside University, Middlesbrough, United Kingdom; ^4^ Department of Pharmacology and Toxicology, College of Pharmacy, King Saud University, Riyadh, Saudi Arabia

**Keywords:** weight-for-height, growth charts, height-for-age, Jordan, weight-for-age

## Abstract

**Purpose:**

This study aimed to determine the prevalence of underweight, overweight, and obesity among Jordanian infants aged 0–2 years, establish national growth reference charts, and compare the growth of Jordanian infants with the WHO growth standards.

**Methods:**

The present study analyzed 260,027 anthropometric measurements derived from 82,874 healthy Jordanian children (51% boys) aged 0–24 months. These measurements included both cross-sectional and repeated entries, with each child contributing between one visit and nine follow-up visits (10 measurements). Weight and height measurements were analyzed using the Generalized Additive Models for Location Scale and Shape (GAMLSS) statistical method to develop the growth charts.

**Results:**

Separate models for height-for-age, weight-for-age, and weight-for-height were constructed for each gender. Significant discrepancies were found between WHO growth references and the Jordanian references. Children in Jordan were shorter, particularly among girls, and had slightly higher weight-for-age from the age of 7 months onward.

**Conclusion:**

The availability of Jordanian-specific growth references will improve the accuracy of assessing children's growth and enhance the monitoring and evaluation of their health and development.

## Introduction

The optimal growth of children plays a pivotal role in their overall well-being and development, as it is closely linked to their overall health (1). Monitoring children's growth serves multiple purposes, including alleviating concerns regarding atypical development, facilitating comprehensive progress, and identifying underlying health conditions (2). Various methods are employed for evaluating children's growth, with one such approach involving the longitudinal assessment of height and weight changes, providing a straightforward way to compare against established growth references and identify developmental trends (3).

In April 2006, the World Health Organization (WHO) released updated growth curves derived from the Multicenter Growth Reference Study (MGRS), conducted between 1997 and 2003 in Brazil, Ghana, India, Norway, Oman, and the USA (4). According to the WHO, breastfed infants and children from economically advantaged backgrounds exhibit similar patterns of unrestricted growth. Consequently, a unified set of growth curves can effectively represent typical human physiology up to the age of 5 (3). Recognized as growth references, these curves serve as a universal benchmark for growth, applicable across all nations, regardless of genetic or cultural diversity. Any deviations from these references may indicate abnormal growth patterns (3). However, while WHO growth standards depict a child's growth in ideal circumstances, numerous disparities, such as economic, social, and environmental factors, impede the growth and development of children in many regions of the world where optimal growth conditions are not prevalent (5).

Currently, public maternity and child health institutions in Jordan use the 2006 WHO growth charts to monitor children's growth. However, differences in children's body size and shape among populations stem from variations in genetic makeup, environmental factors, and their interplay (6). This has been shown in previous studies conducted in Jordan which found significant disparities between healthy Jordanian growth patterns and WHO growth standards (7, 8). The evident misclassification of anthropometric measurements resulting from reliance on international references in Jordanian pediatric healthcare facilities underscores the impracticality of using them for assessing the growth of children within the Jordanian population (7, 8).

Several countries have recognized the limitations of applying international growth standards across diverse populations and have developed localized growth references. For example, studies from Pakistan have reported substantial differences in growth patterns among school-aged children and adolescents compared to WHO references, which could lead to the misclassification of healthy individuals as undernourished (9, 10). In Indonesia, the use of international charts was shown to overestimate the prevalence of stunting and wasting among children, particularly in rural areas (11). Similarly, Turkish head circumference percentiles only partially aligned with U.S. and Belgian norms, highlighting the value of national reference data (12). More recently, Iran and Syria have also produced country-specific growth references, aiming to provide more accurate and contextually relevant standards for child health monitoring (13, 14). Together, these findings underscore the growing international consensus that local growth charts are needed to account for ethnic, environmental, and nutritional factors affecting child development.

Additional examples of national efforts to replace WHO or CDC references can be in North Africa. Egypt has developed *Z*-score growth references for both preschool-aged children and school-aged children up to 19 years, using nationally representative samples and LMS methodology (15, 16). These efforts reflect a growing regional trend toward context-specific solutions that better account for local genetic, nutritional, and environmental influences.

This highlights the urgent need to create a dedicated national growth chart tailored specifically to Jordanian children aged 0–2 years. This study specifically focuses on developing growth charts for Jordanian children aged 0–2 years. This age group was prioritized due to it being a period of exceptionally rapid physical growth and neurodevelopment, making it a critical window for health monitoring and nutritional interventions. Furthermore, the growth dynamics during these first 2 years are distinct from later childhood, warranting focused modeling to accurately capture these unique trajectories, an approach also reflected by the WHO MGRS that had a distinct longitudinal component that followed children from birth to 24 months (3). The availability of a robust and extensive dataset for this age ranges from the national Hakeem program also enabled a detailed and well-powered analysis for this crucial developmental stage. Finally, the clinical relevance is high, as healthcare visits and growth monitoring are most frequent in these first 2 years, making tailored charts for this period a valuable tool for Jordanian healthcare professionals for early detection and management of growth issues. This chart will include crucial measurements such as weight-for-age (WFA), length-for-age (LFA), and weight-for-length (WFL). By customizing these growth indicators to suit the distinct characteristics and growth patterns of Jordanian children, this endeavor aims to offer healthcare professionals and researchers a more accurate and culturally appropriate resource for tracking and assessing children's growth and development within the country. The study also aimed to assess the prevalence of underweight, overweight, and obesity among Jordanian infants aged 0–2 years and to compare their growth with the WHO growth standards.

## Methods

### Data collection

A mixed-model, cross-sectional and repeated-measures study was conducted across three key regions of Jordan—the north, central, and south. A stratified sampling approach was applied during data extraction to ensure that the regional distribution of the sample closely mirrored that of the target population (Supplementary Table S1). The data were stratified by location, sex, and measurement year. Data were collected through Jordan's national e-health program, known as the Hakeem program, which utilizes electronic health records. This program plays a pivotal role in Jordan's healthcare system, offering invaluable data resources pertinent to this study. The dataset includes anthropometric measurements of children aged 0–2 years, aligning with established WHO standards (3). Comprehensive and representative data were sourced from 115 maternal and child health centers nationwide, spanning from April 2016 to June 2023. This dataset includes demographic details such as gender, date of birth, and visit date, enabling estimation of the child's age at the time of measurement. Additionally, actual physical measurements derived from the hospital records, such as weight and recumbent length, were documented. Furthermore, data on the health status and medication history of each toddler were collected to identify potential factors impacting their growth and development. Ethical approval was obtained from the Institutional Review Board (IRB) via Letter No. IRB-02/581/2021-2/2022 dated 13/06/2022, with a waiver of informed consent since there was no direct interaction with human subjects in this study.

### Data cleaning

The initial sample collected from the Hakeem program comprised 272,199 anthropometric measurements of full-term children born within the gestational age range of 37–42 weeks with available full medical profile. Exclusion criteria were meticulously applied to ensure the representativeness of growth patterns observed among Jordanian children. Only records from children considered healthy at the time of measurement were included. Health status was evaluated based on available clinical data, and children with chronic or acute conditions known to impact growth (e.g., congenital heart defects, cystic fibrosis, chronic renal disease) those on long-term medications affecting growth (e.g., corticosteroids), congenital anomalies, or preterm birth were excluded. However, data on breastfeeding status, maternal health, or environmental exposures (e.g., smoking during pregnancy) were not available in the dataset and could not be incorporated into the inclusion criteria. Yet, the study aim was to develop growth references representative of the general healthy Jordanian child population as captured in the national health system, rather than prescriptive standards based on optimized conditions like the WHO MGRS (3). Applying these selection criteria led to the exclusion of 11,368 records who were either born prematurely or diagnosed with medical conditions that could potentially affect their growth. The objective was to secure a sample consisting solely of healthy children devoid of underlying medical influences on growth trajectories. Additionally, 498 outliers were identified through *z*-scores for length and weight, signifying observations deviating from the anticipated range for the population. Typically, such outliers stem from inaccuracies in data entry or measurement rather than extraordinary growth (4). A fixed exclusion range, guided by the 1977 National Center for Health Statistics and World Health Organization (NCHS/WHO) guidelines, was applied to identify and eliminate outliers (4). Any *z*-score values falling outside this range were disregarded, with specific exclusion thresholds set for LFA (<−5 and >+3), WFA (<−5 and >+5), and WFL (<−4 and >+5). Furthermore, duplicate entries, unavailable or missing data, and biologically implausible values (BIVs) were meticulously scrutinized and excluded from the analysis, resulting in the elimination of 306 observations. Following rigorous data cleaning and the removal of biologically implausible values, the final dataset included 260,027 anthropometric records collected from 84,354 unique healthy children (41,284 girls and 43,070 boys) (Supplementary Figure A1). Each child contributed between one and ten measurements, reflecting both cross-sectional and repeated entries. A detailed breakdown of follow-up frequencies per child is provided in Supplementary Table S2 in the Supplementary. The age at which measurements were taken was calculated in days and sorted into age intervals.

### Statistical analysis

SPSS version 23 and RStudio version 4.2.764 with GAMLSS package version 5.4–12 were used to analyze the data. Continuous data were presented as means. The LFA, WFA, and WFL data were fitted to the Box-Cox power exponential (BCPE) distribution, which provides a suitable modeling approach for variables that deviate from linearity and normality assumptions. In growth charts where skewness and kurtosis are present and normality cannot be assumed, the BCPE distribution accurately captures the underlying patterns of child growth (17).

The BCPE distribution is characterized by four parameters denoted as *μ*, *σ*, *ν*, and *τ*, representing location (median), scale (approximate coefficient of variation), skewness (transformation to symmetry), and kurtosis (power exponential parameter), respectively. The BCPE distribution ultimately simplified to the Lambda-Mu-Sigma (LMS) method (18), where lambda captures skewness, mu represents the median, and sigma represents the coefficient of variation. In line with the methodology adopted by the WHO MGRS (3), repeated measures and cross-sectional records were appended into a single analytic file. All valid anthropometric records were modeled together using the LMS method, without stratification by measurement frequency. This approach allows the inclusion of both single and repeated measurements per child while preserving the integrity of the reference curves. This enables the construction of growth charts and percentile curves that effectively capture the evolving patterns of child growth and assess different aspects of child development. Furthermore, the LMS method does not impose a particular distribution requirement, offering flexibility in modeling various distributional shapes. This includes skewed distributions, exponential distributions, normal distributions (Gaussian distributions), and numerous others. Additionally, the LMS method allows for the incorporation of smoothing splines, which enhance the model's fitness to the observed data.

In the model selection process, spline smoothing techniques were employed to estimate the parameters of skewness, median, sigma, and kurtosis. The choice of the smoothing method was based on its ability to capture the underlying growth patterns without overfitting (19). The Akaike Information Criterion (AIC), measures of goodness-of-fit (GD), and degrees of freedom (df) were utilized to compare different models and determine the best fit for the Jordanian data.

In constructing the Jordanian growth references, this study adhered to the WHO guidelines (3). The modeling process began with a transformation of the age or length variable to improve model flexibility and fit, particularly during early life, where growth is most dynamic. A power transformation (e.g., age^*λ* or length^*λ*) was applied, and the optimal transformation exponent (*λ*) was selected based on reductions in GD across a pre-specified grid. Using this transformed scale, initial models were fitted for the median (μ) and coefficient of variation (*σ*) parameters using the Box-Cox Power Exponential (BCPE) distribution within a GAMLSS framework, with penalized cubic splines providing smooth functions of age or length. At this stage, skewness (*ν*) and kurtosis (*τ*) were fixed at default values (*ν* = 1, *τ* = 2), reducing the model to a simplified LMS form. The selection of degrees of freedom (df) for the μ and *σ* curves was based on minimizing the Akaike Information Criterion (AIC) and its generalized form GAIC (3), while ensuring biological plausibility and smoothness of the resulting centile curves. Once μ and *σ* were optimized, the skewness parameter *ν* was modeled with its own spline, and degrees of freedom were again selected based on GAIC (3) and centile comparisons. Modeling of the kurtosis parameter (*τ*) was only considered when residual diagnostics revealed significant deviation from normality in the distribution tails. The entire process was iterative, with each adjustment prompting reevaluation of previously fixed parameters and model structure. Validation included local and global goodness-of-fit assessments, residual diagnostics, and empirical checks of centile coverage across the age range.

Once the optimal equations for LFA, WFA, and WFL had been determined separately for boys and girls, the corresponding values of median, sigma, skewness, and kurtosis were identified for each model. To ensure a comprehensive analysis, suitable smoothing splines were applied to account for age variations in each parameter. This approach facilitated the construction of sex-specific growth charts for each parameter, ensuring an accurate depiction of growth patterns over different ages.

## Results

The descriptive statistics in Table 1 offer a comprehensive summary of anthropometric measures across age intervals for both boys and girls. Among boys, a clear growth trajectory is observed, with mean height progressing from 51.7 cm (SD = 2.39) in the 0–44-day group to 86.4 cm (SD = 4.12) in those aged 720 days or more. Correspondingly, mean weight increases from 3.94 kg (SD = 0.58) to 12.5 kg (SD = 1.42). Similarly, among girls, mean height grows from 50.9 cm (SD = 2.30) in the youngest group to 84.2 cm (SD = 3.61) in the oldest, with weight increasing from 3.7 kg (SD = 0.53) to 11.7 kg (SD = 1.13). While the growth patterns are broadly similar between sexes, boys generally exhibit slightly higher mean values in both height and weight, particularly in the upper age ranges.

**Table 1:** Descriptive statistics of length and weight by age group (boys).

Age group (days)	*N*	Length mean (SD)	Weight mean (SD)	*N*	Length mean (SD)	Weight mean (SD)
	Boys (*N* = 132,423)			Girls (*N* = 127,604)		
0–44	17,577	51.7 (2.39)	3.94 (0.58)	16,563	50.9 (2.30)	3.7 (0.53)
45–89	17,533	58.2 (2.40)	5.63 (0.64)	16,780	57.0 (2.37)	5.2 (0.59)
90–134	21,357	62.2 (2.55)	6.66 (0.73)	20,185	60.7 (2.50)	6.1 (0.68)
135–179	13,016	65.1 (2.52)	7.4 (0.78)	12,572	63.5 (2.44)	6.8 (0.71)
180–224	5,158	67.8 (2.54)	8.09 (0.87)	4,964	66.0 (2.49)	7.5 (0.81)
225–269	2,112	70.0 (2.62)	8.68 (0.93)	2,128	68.2 (2.58)	8.0 (0.88)
270–314	18,138	72.4 (2.56)	9.36 (0.91)	17,638	70.9 (2.62)	8.7 (0.90)
315–359	3,255	73.7 (2.67)	9.6 (0.93)	3,379	72.3 (2.71)	9.0 (0.93)
360–404	15,781	76.0 (2.72)	10.2 (0.98)	15,346	74.6 (2.75)	9.6 (0.96)
405–449	2,112	77.3 (2.73)	10.5 (0.99)	2,197	76.0 (2.82)	9.9 (1.00)
450–494	680	78.7 (3.08)	10.7 (1.11)	600	77.3 (2.97)	10.1 (1.00)
495–539	562	81.1 (3.17)	11.3 (1.12)	578	79.8 (3.18)	10.6 (1.17)
540–584	9,893	82.2 (2.94)	11.6 (1.08)	9,653	80.9 (2.90)	11.0 (1.07)
585–629	3,313	83.1 (3.06)	11.8 (1.09)	3,288	82.1 (3.03)	11.3 (1.09)
630–674	1,253	84.0 (3.20)	12.0 (1.09)	1,065	83.0 (3.25)	11.5 (1.16)
675–719	551	85.2 (3.54)	12.3 (1.25)	568	84.1 (3.39)	11.7 (1.18)
≥720	132	86.4 (4.12)	12.5 (1.42)	100	84.2 (3.61)	11.7 (1.13)

### Creation of Jordan-specific growth charts

In order to provide a comprehensive framework for constructing growth charts that accurately represent the distribution of measurements and account for age- and gender-specific characteristics, the BCPE distribution was used to capture the distribution of data by age and gender. The produced models' specifications are displayed in Table 2.

**Table 2:** Summary of GAMLSS model parameters, degrees of freedom (df), and goodness-of-fit statistics.

Model description	Specified df for μ smoother	Specified df for *σ* smoother	Specified df for *ν* smoother	Specified df for *τ* smoother	Total model edf	global deviance	AIC	SBC
Weight-for-age (boys)	16	16	12	8	40.42	301,358.0	301,438.9	301,834.7
Height-for-age (boys)	14	18	16	6	52.21	641,221.8	641,326.3	641,840.1
Weight-for-height (boys)	20	16	22	16	71.77	254,779.4	254,922.9	255,622.7
Height-for-age (girls)	12	12	14	8	56.49	617,526.0	617,639.0	618,192.9
Weight-for-length (girls)	24	22	20	2	52.58	255,525.3	255,641.3	256,206.9
Weight-for-age (boys)	22	14	8	18	55.67	277,417.7	277,529.1	278,072.2

### Length-for-age (LFA)

**Figure 1:**
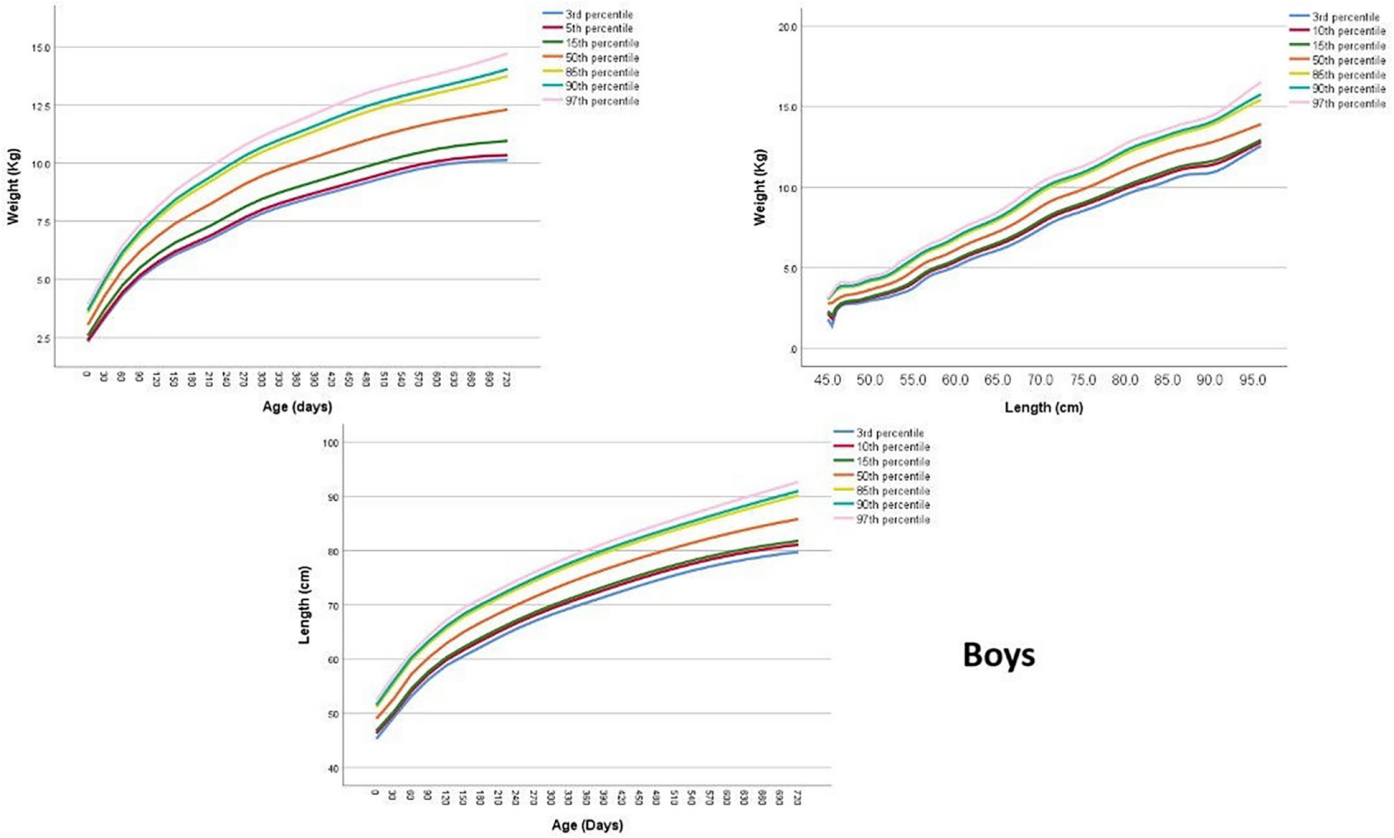
Length-for-age, weight-for-age and weight-for-length centile curves at 3rd 15th 50th 85th and 97th centiles for Jordanian boys from 0 to 2 years.

**Figure 2:**
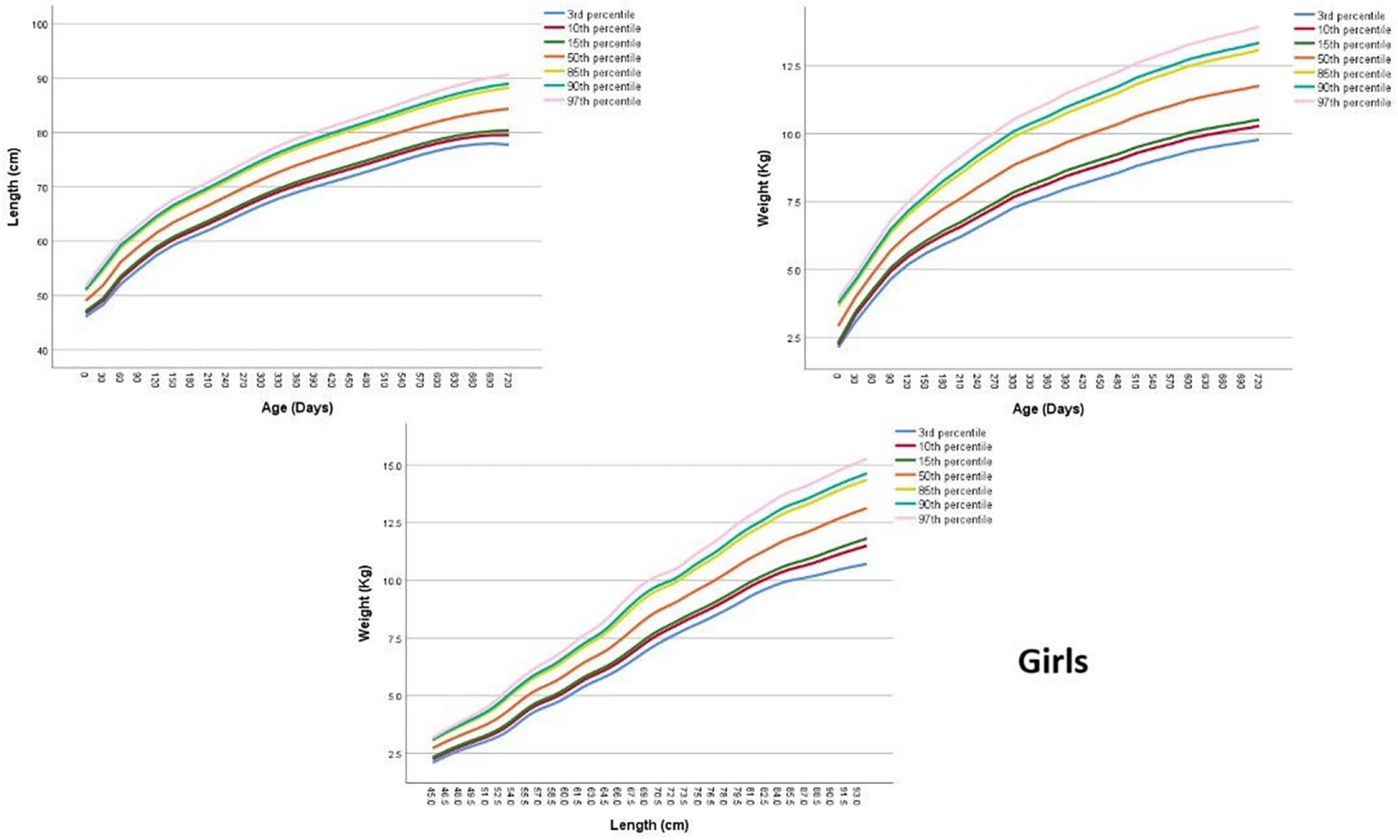
Length-for-age, weight-for-age and weight-for-length centile curves at 3rd 15th 50th 85th and 97th centiles for Jordanian girls from 0 to 2 years.

Upon application of the LFA model, values of the specific LFA parameters (μ, *σ*, *ν*, *τ*) for children aged between 0 and 2 years were directly obtained. The resulting LFA values for boys and girls are presented in Supplementary Table S3 in the supplementary material, with corresponding values for each age. Subsequently, the models were interpolated to estimate centiles and SDs at each age (Supplementary Tables S3, S4). Visual representations of the centiles are provided in Figures 1, 2, showcasing the centiles specifically for boys and girls, respectively.

### Weight-for-age (WFA)

Supplementary Table S3 in the supplementary material presents the values of the specific WFA parameters (μ, *σ*, *ν*, *τ*) for boys and girls aged between 0 and 2 years. To further understand the distribution of the data, Figures 1, 2; Supplementary Table S3 provides the estimated centiles and SDs for boys and girls, while estimated SDs are presented in Supplementary Table S4.

### Weight-for-length (WFL)

Supplementary Table S5 presents the values of the specific WFL parameters (μ, *σ*, *ν*, *τ*) for boys and girls. These tables provide valuable information about the specific WFL parameters for each gender. To further understand the distribution of the data, Figures 1, 2; Supplementary Table S5 depict the estimated centiles and Supplementary Table S6 shows the estimated SDs for each length in boys and girls.

### Comparison with WHO

**Figure 3:**
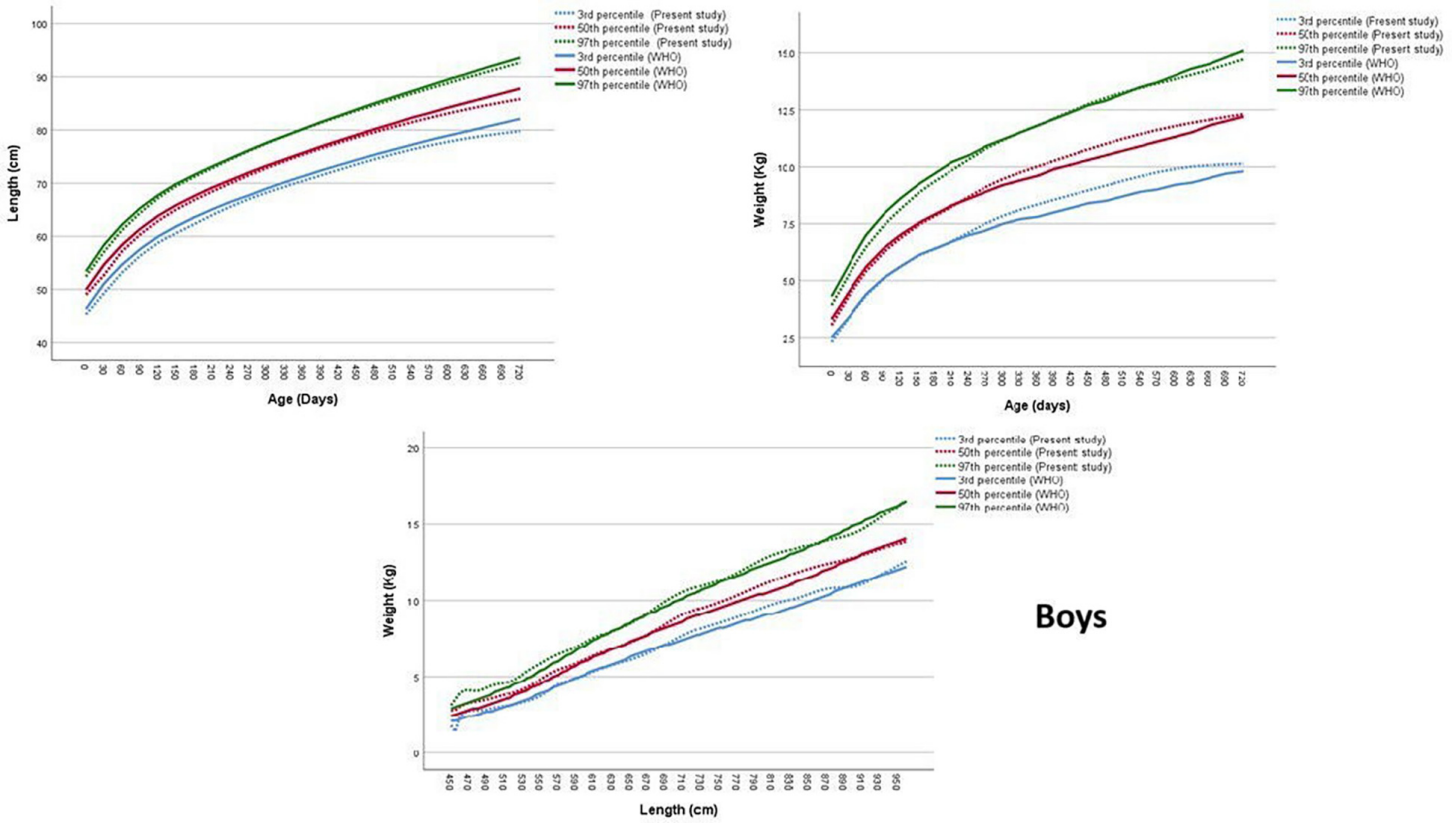
Comparison of the length-for-age, weight-for-age and weight-for-length 50th centile between Jordan and WHO equations, boys.

**Figure 4:**
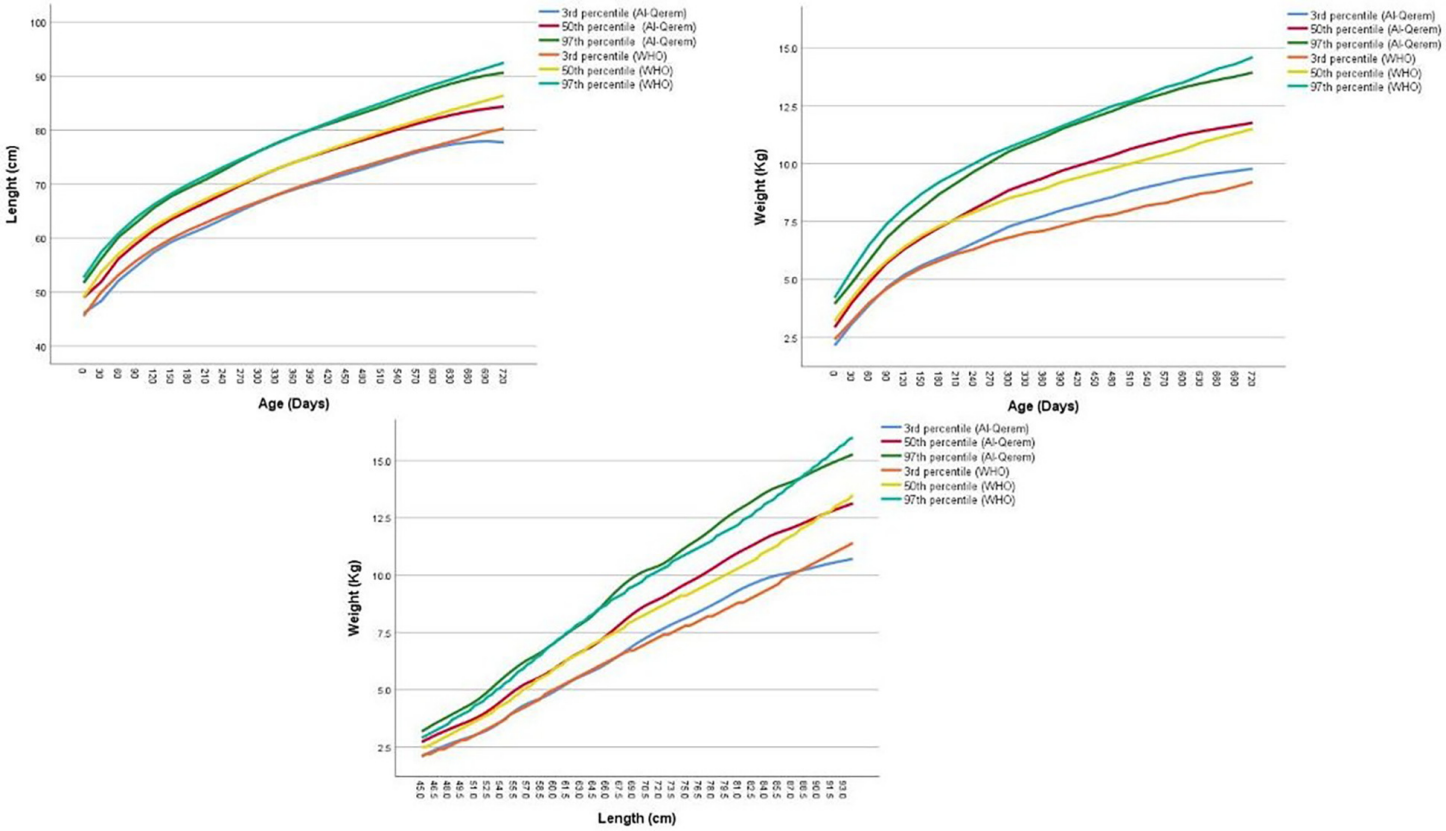
Comparison of the length-for-age, weight-for-age and weight-for-length 50th centile between Jordan and WHO equations, girls.

The comparative data in Supplementary Tables S7, S8; Figures 3, 4 reveal important differences in early childhood growth patterns between Jordanian reference and the WHO reference, both in terms of absolute centile values and the breadth of the centile span. For LFA, across nearly all centiles (3rd, 10th, 50th, 90th, and 97th), the WHO standards consistently report slightly higher values than the Jordanian references at most age points for both boys and girls. This trend persists throughout the first two years of life, with the difference most pronounced at the upper percentiles (e.g., 90th and 97th), where the WHO standards show taller children compared to their Jordanian peers. The span between the 3rd and 97th percentiles, the range that captures the vast majority of normal growth, also tends to be slightly wider in the WHO standards, indicating greater variability.

In contrast, WFA centiles display a more subtle pattern. At birth and during the earliest months, the WHO standards again report marginally higher weights at each corresponding percentile, indicating that infants in the WHO sample may be heavier at birth and in early infancy. However, as age increases, particularly beyond 6 months, the Jordanian WFA centiles begin to converge with or even surpass the WHO references at certain percentiles and age intervals. This is especially evident at the upper centiles (90th and 97th) in boys, where Jordanian children may demonstrate comparable or slightly greater weight values, suggesting a potential shift in growth velocity or patterns of weight gain after infancy.

The centile span tends to be broader in the WHO standards for both LFA and WFA, particularly for boys. This wider range reflects a more pronounced spread in the distribution of height and weight values, possibly due to greater heterogeneity in the WHO reference sample. Among Jordanian children, the centile span is slightly narrower, which may indicate more homogeneity in growth patterns within the local population.

The comparison of WFL centiles between Jordanian references and WHO standards reveals distinct patterns in both boys and girls, with important implications for clinical assessment and nutritional interpretation. For boys, a comparison of the median values indicates subtle differences between the Jordanian references and WHO standards. At shorter lengths, such as 45.0 cm, the Jordanian median weight is slightly higher than the WHO median (3.8 vs. 2.4 kg). This trend of slightly higher or comparable median weights for the Jordanian cohort continues for lengths up to around 60.0 cm. beyond that length, this pattern is fluctuating, for example at 80.0 cm, the Jordanian median (is slightly higher than the WHO median (11.0 vs. 10.4 kg), but at 95.0 cm, the WHO median slightly surpasses the Jordanian median (13.9 vs. 13.7 kg). The span of distribution, assessed by the difference between the 3rd and 97th percentiles, generally increases with length for both Jordanian references and WHO standards in boys. This suggests that the variability in weight tends to be greater for longer boys. The WHO span is narrower at the shortest length (45.0 cm) but becomes slightly wider than the Jordanian span at the longest length (95.0 cm).

For girls, an examination of median values reveals that Jordanian reference weights are frequently higher than WHO standards across most lengths measured from 45.0 cm to 90.0 cm. For instance, at a length of 45.0 cm, the Jordanian median weight is 2.72 kg, compared to the WHO median of 2.50 kg. However, there are points of near convergence, such as at 60.0 cm where both medians are 5.90 kg, and a slight reversal at 65.0 cm where the Jordanian median (7.06 kg) is marginally lower than the WHO median (7.10 kg). The span of distribution, representing the difference between the 3rd and 97th percentiles, consistently widens with increasing length for both Jordanian references and WHO standards, suggesting an increased variability in weight as girls grow longer. Comparing these spans, the Jordanian distribution is wider at the shortest length of 45.0 cm (1.08 kg for Jordanian vs. 0.80 kg for WHO). At the longest length of 90.0 cm, the Jordanian span (4.22 kg) is also slightly wider than the WHO span (4.20 kg).

The prevalence of WFL nutritional status categories was evaluated using both WHO standards and model-derived residuals based on the Jordan-specific growth equation, separately for boys and girls. Among boys, the WHO reference identified 6.6% as overweight (*z*-score > + 2 SD) and 1.95% as underweight (*z*-score ≤2 SD). When assessed using residuals from the Jordanian equation, the estimated prevalence declined to 2.25% for overweight and increased slightly to 2.71% for underweight. Among girls, the WHO criteria classified 6.06% as overweight and 1.67% as underweight. Using the Jordan-specific residuals, the corresponding proportions were 2.61% and 2.59% for overweight and underweight, respectively (Tables 3, 4).

**Table 3:** Distribution of nutritional status by age group and growth reference for boys.

Age group	Jordan			WHO		
	Obese	Overweight	Underweight	Obese	Overweight	Underweight
0–29 days	0 (0%)	143 (1%)	542 (3.8%)	413 (2.9%)	1,910 (13.4%)	485 (3.4%)
30–59 days	0 (0%)	90 (2.1%)	99 (2.3%)	116 (2.7%)	531 (12.4%)	124 (2.9%)
60–89 days	0 (0%)	265 (1.6%)	613 (3.7%)	182 (1.1%)	1,309 (7.9%)	679 (4.1%)
90–119 days	0 (0%)	300 (2.1%)	357 (2.5%)	43 (0.3%)	656 (4.6%)	499 (3.5%)
120–149 days	0 (0%)	341 (2.5%)	314 (2.3%)	14 (0.1%)	437 (3.2%)	410 (3%)
150–179 days	0 (0%)	161 (2.5%)	200 (3.1%)	0 (0%)	187 (2.9%)	187 (2.9%)
180–209 days	0 (0%)	88 (2.3%)	84 (2.2%)	0 (0%)	111 (2.9%)	65 (1.7%)
210–239 days	0 (0%)	56 (2.6%)	54 (2.5%)	0 (0%)	88 (4.1%)	28 (1.3%)
240–269 days	0 (0%)	32 (2.4%)	37 (2.8%)	0 (0%)	66 (5%)	7 (0.5%)
270–299 days	0 (0%)	327 (2.3%)	327 (2.3%)	0 (0%)	910 (6.4%)	14 (0.1%)
300–329 days	0 (0%)	139 (2.4%)	139 (2.4%)	0 (0%)	354 (6.1%)	12 (0.2%)
330–359 days	0 (0%)	36 (2.6%)	47 (3.4%)	0 (0%)	77 (5.6%)	0 (0%)
360–389 days	0 (0%)	335 (2.5%)	308 (2.3%)	0 (0%)	696 (5.2%)	13 (0.1%)
390–419 days	0 (0%)	76 (2.2%)	87 (2.5%)	0 (0%)	174 (5%)	0 (0%)
420–449 days	1 (0.1%)	32 (3.1%)	30 (2.9%)	0 (0%)	63 (6.1%)	0 (0%)
450–479 days	0 (0%)	13 (2.5%)	25 (4.9%)	0 (0%)	22 (4.4%)	0 (0%)
480–509 days	1 (0.3%)	7 (2.4%)	7 (2.1%)	0 (0%)	16 (5.2%)	0 (0%)
510–539 days	0 (0%)	8 (2%)	9 (2.2%)	0 (0%)	25 (5.9%)	0 (0%)
540–569 days	0 (0%)	227 (3.1%)	139 (1.9%)	0 (0%)	556 (7.6%)	7 (0.1%)
570–599 days	4 (0.1%)	137 (3.3%)	70 (1.7%)	0 (0%)	290 (7%)	12 (0.3%)
600–629 days	2 (0.1%)	58 (3.3%)	51 (2.9%)	0 (0%)	117 (6.7%)	11 (0.6%)
630–659 days	0 (0%)	37 (4%)	17 (1.8%)	0 (0%)	52 (5.6%)	7 (0.8%)
660–689 days	0 (0%)	23 (4.1%)	13 (2.3%)	0 (0%)	30 (5.4%)	8 (1.4%)
690–719 days	0 (0%)	13 (4.2%)	11 (3.5%)	0 (0%)	24 (7.6%)	2 (0.7%)
720–749 days	0 (0%)	6 (4.2%)	2 (1.7%)	0 (0%)	4 (3.3%)	0 (0%)

**Table 4:** Distribution of nutritional status by age group and growth reference for girls.

Age group	Jordan			WHO		
	Obese	Overweight	Underweight	Obese	Overweight	Underweight
0–29 days	0 (%)	276 (2%)	442 (3.2%)	0 (%)	1,201 (8.7%)	400 (2.9%)
30–59 days	0 (%)	127 (3.3%)	93 (2.4%)	0 (%)	397 (10.3%)	73 (1.9%)
60–89 days	0 (%)	333 (2.1%)	397 (2.5%)	0 (%)	1,143 (7.2%)	413 (2.6%)
90–119 days	0 (%)	328 (2.4%)	369 (2.7%)	0 (%)	560 (4.1%)	437 (3.2%)
120–149 days	0 (%)	311 (2.4%)	350 (2.7%)	0 (%)	350 (2.7%)	376 (2.9%)
150–179 days	0 (%)	171 (2.8%)	140 (2.3%)	0 (%)	183 (3%)	140 (2.3%)
180–209 days	0 (%)	81 (2.3%)	92 (2.6%)	0 (%)	106 (3%)	74 (2.1%)
210–239 days	0 (%)	73 (3.4%)	50 (2.3%)	0 (%)	103 (4.8%)	28 (1.3%)
240–269 days	0 (%)	35 (2.7%)	35 (2.7%)	0 (%)	68 (5.3%)	14 (1.1%)
270–299 days	0 (%)	445 (3.2%)	333 (2.4%)	0 (%)	847 (6.1%)	69 (0.5%)
300–329 days	0 (%)	161 (2.8%)	161 (2.8%)	0 (%)	310 (5.4%)	29 (0.5%)
330–359 days	0 (%)	29 (2.1%)	34 (2.4%)	0 (%)	69 (4.9%)	6 (0.4%)
360–389 days	0 (%)	370 (2.8%)	318 (2.4%)	0 (%)	794 (6%)	13 (0.1%)
390–419 days	0 (%)	106 (3.2%)	90 (2.7%)	0 (%)	239 (7.2%)	10 (0.3%)
420–449 days	0 (%)	28 (2.9%)	26 (2.7%)	0 (%)	72 (7.4%)	0 (0%)
450–479 days	0 (%)	14 (3.4%)	14 (3.2%)	0 (%)	36 (8.4%)	1 (0.2%)
480–509 days	0 (%)	8 (2.7%)	11 (3.7%)	0 (%)	21 (7%)	0 (0%)
510–539 days	0 (%)	12 (2.8%)	13 (3%)	0 (%)	28 (6.3%)	2 (0.5%)
540–569 days	0 (%)	169 (2.3%)	176 (2.4%)	0 (%)	595 (8.1%)	7 (0.1%)
570–599 days	0 (%)	126 (3.2%)	94 (2.4%)	0 (%)	358 (9.1%)	8 (0.2%)
600–629 days	0 (%)	47 (2.8%)	40 (2.4%)	0 (%)	145 (8.6%)	8 (0.5%)
630–659 days	0 (%)	23 (2.9%)	12 (1.5%)	0 (%)	74 (9.5%)	4 (0.5%)
660–689 days	0 (%)	14 (3%)	6 (1.3%)	0 (%)	36 (7.8%)	2 (0.4%)
690–719 days	0 (%)	10 (2.7%)	8 (2.1%)	0 (%)	24 (6.8%)	3 (0.9%)
720–749 days	0 (%)	2 (2.5%)	2 (2.5%)	0 (%)	5 (5%)	1 (0.8%)

### Model fitness

The quantile residual diagnostics of the generated models provide compelling evidence of the statistical adequacy of the six fitted GAMLSS models for both sexes (Supplementary Table S9). Across all models, residual means hovered tightly around zero, suggesting unbiased prediction. Residual variances were close to 1, indicating proper model calibration without evidence of overdispersion or under dispersion. Skewness values were consistently near zero, signifying residual symmetry, while kurtosis estimates remained close to the expected value of 3, reflecting appropriate tail behavior.

Filliben correlation coefficients ranged from 0.9985 to 0.9994 across all models, strongly supporting the assumption of approximate normality of residuals. Notably, both weight-for-height models (boys: 0.9994; girls: 0.9993) achieved the highest normality correlations, highlighting their particularly well-fitting behavior across centiles.

The centile coverage evaluations for both female and male pediatric populations provide compelling evidence of the strong calibration and reliability of the fitted GAMLSS models in representing growth trajectories (Supplementary Table S10). Among girls, the height-for-age and WFA models showed excellent agreement with theoretical centile expectations, with deviations across percentiles remaining narrow and generally within ±1.5%. Notably, the 50th percentile was almost perfectly centered in both models, suggesting accurate median estimation. The WFL model similarly demonstrated robust calibration, aside from a modest overestimation at the 25th percentile. In parallel, the male models displayed similarly strong performance. For height-for-age and WFA, the observed centile proportions aligned closely with their theoretical counterparts, particularly at the 3rd, 50th, and 97th percentiles. The weight-for-height model also showed tight correspondence across the distribution, with minor deviations that did not exceed 1.5%.

## Discussion

Since the eighteenth century, the study of human growth patterns has captivated human biologists and public health experts (20). This comprehensive study, based on a large population, provides growth benchmarks for Jordanian children aged 0–24 months in terms of LFA, WFA, and WFL.

The charts for LFA, WFA, and WFL were derived from a comprehensive sample of 82,874 children (51% boys), comprising 260,027 anthropometric measurements (50.9% boys). These children attended maternal and child health centers across the three primary regions in Jordan (north, central, and south). This diverse representation across gender, age groups, and geographic areas enhances the reliability of our findings. Our meticulous data analysis provides precise insights into the growth patterns of children in Jordan, offering valuable implications for healthcare providers and policymakers, particularly in nutrition and growth monitoring. Tailored interventions may be warranted to foster healthy growth among Jordanian boys and girls.

These growth references are provided in two formats: centiles/SDs and LMS (Lambda-Mu-Sigma) curves based on the BCPE (Box-Cox Power Exponential) method. The classic centile/SDs charts and tables are user-friendly and can be easily interpreted by parents and healthcare providers, offering a straightforward understanding of a child's growth relative to peers. In contrast, the LMS values are particularly useful for auxologists and researchers, as they allow for the detection and analysis of more nuanced growth characteristics, such as skewness and kurtosis, which may be present in the data (17). This dual approach ensures that both practical and analytical needs are met, enhancing the overall utility of the growth references.

The comparative analysis of Jordanian and WHO growth references reveals systematic and clinically pertinent differences in early childhood growth trajectories. A key observation is that WHO LFA standards consistently position children as taller than Jordanian references across most ages and centiles for both boys and girls. This implies that employing WHO LFA charts in a Jordanian context might lead to a higher apparent prevalence of shortness or stunting if the normative linear growth in Jordanian children naturally follows a slightly lower trajectory than the WHO prescriptive cohort.

In contrast, weight-based metrics such as WFA and WFL exhibit more dynamic patterns. While infants in the WHO sample appear marginally heavier at birth and in the initial months, Jordanian children, particularly boys at the upper centiles, tend to converge with or even exceed WHO WFA references after approximately six months. This suggests a potential divergence in the tempo or pattern of weight gain post-infancy. This trend is further confirmed by WFL findings, where Jordanian boys at shorter lengths and girls across most lengths often show higher median weights for their respective lengths compared to WHO standards. Such discrepancies indicate that Jordanian children might frequently be classified as heavier or at risk of overweight by WHO WFL criteria, even if their growth is typical for their local population.

Furthermore, the observed differences in the centile spans, generally wider in WHO standards for LFA and WFA, likely reflect the greater heterogeneity inherent in the multinational WHO reference sample. The comparatively narrower spans in the Jordanian data could suggest more homogeneous growth patterns within this specific population. These divergences underscore the critical importance of context in growth assessment. The choice of reference chart can significantly impact the classification of nutritional status, potentially leading to misinterpretation of conditions like stunting, wasting, or overweight, thereby influencing clinical decisions and public health strategies in Jordan. These findings highlight the ongoing discussion about the universal applicability of international growth standards vs. the value of population-specific references that may more accurately reflect local growth characteristics.

Our findings are consistent with those reported in several other middle-income countries where international references tend to overestimate rates of stunting or undernutrition. In Indonesia, children assessed using CDC charts were frequently classified as undernourished, whereas national charts placed the majority within healthy ranges (11). Similar trends have been documented in Pakistan, where local data revealed lower average height and weight compared to WHO standards, but with consistent internal growth patterns (9, 10). In Turkey, national head circumference data also differed from international percentiles, supporting the development of region-specific benchmarks (21). Additionally, Iran and Syria have both developed national growth references to improve the clinical and epidemiological accuracy of growth monitoring in their populations (13, 14). These findings align with our results from Jordan and reinforce the need for population-specific references to avoid erroneous nutritional classifications and support appropriate health policy decisions.

Moreover, our research findings align with previous studies from various countries that have emphasized the importance of considering population-based growth references. For instance, studies from Belgium, the Netherlands, Norway, and Denmark have found that infants in these countries are generally longer than the WHO standards. In contrast, research from China and Iran has shown that infants there tend to be shorter than the WHO standards (22). These findings highlight the variability in growth patterns among different populations, suggesting that genetic, environmental, nutritional, and cultural factors significantly influence growth trajectories (23). Therefore, although the WHO standards offer a valuable global benchmark, it is essential to consider regional differences when evaluating child development. Utilizing population-based growth references can enhance the accuracy and relevance of growth assessments, ensuring that they reflect the specific characteristics and needs of local populations. This approach can lead to more tailored and effective health interventions, ultimately supporting better child health outcomes globally.

The growing international trend toward developing national growth references reflects both scientific and practical concerns. While WHO standards provide a unified global benchmark developed under ideal conditions, they often fail to account for the genetic, environmental, and nutritional diversity seen across populations (3, 5). The evidence discussed previously from other countries shows that using WHO curves can result in the misclassification of healthy children as stunted, wasted, or overweight. This misclassification can lead to unnecessary parental concern or inappropriate clinical interventions. In contrast, population-specific growth charts, such as the ones developed in this study, offer more accurate assessments of typical growth patterns within the Jordanian context. This improves the clinical utility of growth assessments and provides a stronger foundation for public health planning. In settings where healthcare resources are limited, using precise national standards can help prioritize care and direct nutritional interventions more effectively. Therefore, implementing national references is a methodological improvement as well as a practical approach to enhancing child health outcomes and strengthening healthcare decision-making.

### Policy and clinical implications

Our findings have important implications for both clinical practice and national health policy. Clinically, the adoption of national growth references will allow healthcare providers to more accurately identify children with growth abnormalities and reduce the risk of misdiagnosis due to reliance on international charts. This will improve decision-making regarding referrals, interventions, and parental counseling. At the policy level, incorporating Jordan-specific growth charts into national guidelines and health information systems, such as the Hakeem electronic health record, would ensure consistent, evidence-based monitoring of child growth. Training programs for pediatricians, nurses, and community health workers should also be updated to reflect the new standards and promote appropriate use in daily practice. Over time, this could support more targeted nutritional and developmental interventions and contribute to more effective resource allocation in early childhood health programs.

### Strengths and limitations

This study has several important strengths that enhance understanding of infant growth in Jordan. Adherence to WHO criteria and rigorous sampling ensured a well-nourished, geographically representative population, minimizing selection bias. The large, sex-balanced sample improves the reliability of findings and enables subgroup analyses. Excluding children with medical conditions or relevant medications further aligned the cohort with WHO standards. Together, these methodological strengths support the validity of the data and provide an accurate reflection of growth patterns among healthy Jordanian children. Furthermore, similar to the WHO approach, the present sample included both repeated measures and cross-sectional data to allow for a better understanding of the growth patterns.

Nevertheless, a few limitations of the present study need to be acknowledged. First, the adherence to international standards when recording the anthropometric measurements by the different nurses was not independently examined by the authors. However, all the anthropometric measurements were taken by qualified nurses working in maternity health institutions. Additionally, due to the anonymized nature of the dataset, we were unable to identify or control for sibling relationships within the sample. As a result, some children from the same household may have been included, potentially introducing familial clustering. Nevertheless, given the large and diverse nature of the dataset, this effect is minimal. Moreover, the WHO MGRS focused on individual-level eligibility criteria such as “single term birth” to exclude known biological confounders but did not explicitly exclude siblings, provided they met the health and feeding criteria (3). This study approach is thus consistent with that precedent. Another limitation of the present study is the lack of information on factors that may influence children's development, including breastfeeding habits, maternal nutrition status, and smoking practices during pregnancy. These variables may influence children's growth patterns (21, 24, 25). Nevertheless, a survey found that over 90% of infants are breastfed at some point, although most were not exclusively breastfed (26). Furthermore, it was reported that only 6.5% of Jordanian girls are smokers and only 2.1% current cigarette smokers (27). Yet, future research should address these limitations to improve understanding of growth patterns in Jordanian children. This includes independently verifying adherence to anthropometric measurement standards to ensure data accuracy and collecting information on key developmental influences such as breastfeeding practices, maternal nutrition, and prenatal smoking. Incorporating these variables would allow for a more comprehensive assessment of growth and strengthen the evidence base for targeted health interventions.

## Conclusion

Assessing children's growth patterns based on international growth standards, particularly when developed from data of different populations, may lead to inaccurate assessments of children's nutritional status. For instance, shorter Jordanian children may be wrongly classified as underdeveloped. Furthermore, the study's findings confirmed this discrepancy; the use of WHO standards resulted in a considerably higher estimated prevalence of overweight for both boys and girls compared to the newly developed Jordanian charts. Therefore, the application of population-specific growth charts developed in the present study has the potential to improve the early recognition of growth abnormalities or underlying health conditions, which could significantly improve health services provided to Jordanian children.

## Data Availability

The datasets presented in this study can be found in online repositories. The names of the repository/repositories and accession number(s) can be found below: Zenodo https://doi.org/10.5281/zenodo.13257769.

## Supplementary material

Supplementary Figure A1 Flow chart of the sample pre-processing and cleaning.

## References

[1] National Research Council and Institute of Medicine. Chapter 3: Influences on children's health. In: Children's Health, The Nation's Wealth: Assessing and Improving Child Health. Washington, D.C.: The National Academies Press (2004). 10.17226/10886

[2] Aw Hui Yong MM Yap F Seng Lee Y. Growth assessment and monitoring during childhood. Ann Acad Med Singap. (2018) 47(4):149–55. 29777245

[3] World Health Organization. WHO Child Growth Standards: Length/Height-for-Age, Weight-for-Age, Weight-for-Length, Weight-for- Height and Body Mass Index-for Age: Methods and Development. Geneva: World Health Organisation (2006). p. 1–312.

[4] Who Multicentre Growth Reference Study Group. Assessment of differences in linear growth among populations in the WHO multicentre growth reference study. Acta Paediatr Suppl. (2006) 450:56–65. 10.1111/j.1651-2227.2006.tb02376.x 16817679

[5] Ong KK. WHO growth standards—suitable for everyone? Yes. Paediatr Perinat Epidemiol. (2017) 31:463–4. 10.1111/PPE.12396 28841750

[6] Hills AP Byrne NM. An overview of physical growth and maturation. Med Sport Sci. (2010) 55:1–13. 10.1159/000321968 20956856

[7] Bataineh L Al-Qerem W Jarab A Alasmari F Eberhardt J. Significant differences in the length and weight measurements of Jordanian infants compared to the world health organization 2006 growth standards. J Family Community Med. (2024) 31:124–32. 10.4103/JFCM.JFCM_337_23 38800793 PMC11114874

[8] Al-Qerem W Zumot R Jarab A Eberhardt J Alasmari F Hammad A. Evaluating the validity of international standards of height, weight, and body mass Index on Jordanian children and adolescents. Healthcare. (2024) 12:1295. 10.3390/healthcare12131295 38998830 PMC11240996

[9] Long L Hamdani SD Hamdani SMZH Zhuang J Khurram H Hadier SG. Establishing age- and sex-specific anthropometric growth references standards for South Punjab adolescents utilizing the LMS method: findings from the Pakistani population. Front Public Health. (2024) 12:1417284. 10.3389/FPUBH.2024.1417284/BIBTEX PMC11424522 39328999

[10] Shehzad MA Khurram H Iqbal Z Parveen M Shabbir MN. Nutritional status and growth centiles using anthropometric measures of school-aged children and adolescents from Multan district. Arch Pédiatr. (2022) 29:133–9. 10.1016/J.ARCPED.2021.11.010 34955308

[11] Hasibuan SN Djer MM Andarie AA Pulungan AB. International standard growth charts overestimate stunting prevalence in Nabire and Jakarta, Indonesia, compared to the Indonesian national growth chart. Clin Pediatr Endocrinol. (2023) 32:82–9. 10.1297/CPE.2022-0047 PMC10068622 37020697

[12] Elmali F Altunay C Mazicioglu MM Kondolot M Ozturk A Kurtoglu S. Head circumference growth reference charts for Turkish children aged 0–84 months. Pediatr Neurol. (2012) 46:307–11. 10.1016/j.pediatrneurol.2012.02.016 22520352

[13] Heidari-Beni M Kelishadi R. Prevalence of weight disorders in Iranian children and adolescents. Arch Iran Med. (2019) 22(9):511–5. 31679373

[14] Zamlout A Alwannous K Kahila A Yaseen M Albadish R Aleid M Syrian national growth references for children and adolescents aged 2–20 years. BMC Pediatr. (2022) 22:1–15. 10.1186/s12887-022-03331-0 35568936 PMC9107173

[15] El Shafie AM El-Gendy FM Allahony DM Omar ZA Samir MA El-Bazzar AN Establishment of Z score reference of growth parameters for Egyptian school children and adolescents aged from 5 to 19 years: a cross sectional study. Front Pediatr. (2020) 8:548535. 10.3389/fped.2020.00368 PMC7385378 32793521

[16] El Shafie AM El-Gendy FM Allahony DM Hegran HH Omar ZA Samir MA Development of LMS and Z score growth references for Egyptian children from birth up to 5 years. Front Pediatr. (2021) 8:598499. 10.3389/fped.2020.598499 33537262 PMC7849193

[17] Rigby RA Stasinopoulos DM. Smooth centile curves for skew and kurtotic data modelled using the box-cox power exponential distribution. Stat Med. (2004) 23:3053–76. 10.1002/SIM.1861 15351960

[18] Cole TJ Green PJ. Smoothing reference centile curves: the LMS method and penalized likelihood. Stat Med. (1992) 11:1305–19. 10.1002/SIM.4780111005 1518992

[19] Wood SN Pya N Säfken B. Smoothing parameter and model selection for general smooth models. J Am Stat Assoc. (2016) 111:1548–63. 10.1080/01621459.2016.1180986/SUPPL_FILE/UASA_A_1180986_SM0752.PDF

[20] Cole TJ. The development of growth references and growth charts. Ann Hum Biol. (2012) 39(5):382–94. 10.3109/03014460.2012.694475 22780429 PMC3920659

[21] Ziegler EE. Growth of breast-fed and formula-fed infants. Nestle Nutr Workshop Ser Pediatr Program. (2006) 58:51–9. 10.1159/000095010 16902325

[22] Natale V Rajagopalan A. Worldwide variation in human growth and the World Health Organization growth standards: a systematic review. BMJ Open. (2014) 4(1):e003735. 10.1136/BMJOPEN-2013-003735 24401723 PMC3902406

[23] Musgrave J. Health and Wellbeing for Babies and Children Contemporary Issues. London: Routledge (2022). p. 182.

[24] Kamiya M Suzuki K Yamagata Z. Effect of maternal active smoking during pregnancy on the trajectory of childhood body mass index: a multilevel analysis using quartiles of birthweight. Tob Induc Dis. (2020) 18:34. 10.18332/TID/119117 32382256 PMC7199658

[25] Al Hadid L Al Rajabi O Alburmawi M. The relationship between iodine nutrition, thyroid function and obstetrical outcomes for Jordanian pregnant women. Jordan J Biol Sci. (2018) 11:285–92.

[26] UNICEF. The Ministry of Heath, WHO and UNICEF Celebrate World Breastfeeding Week in Jordan. UNICEF (2021). Available online at: https://www.unicef.org/jordan/press-releases/ministry-heath-who-and-unicef-celebrate-world-breastfeeding-week-jordan (Accessed May 28, 2025).

[27] The Jordanian Ministry of Health. Jordan National Stepwise Survey (STEPs) for Noncommunicable Diseases Risk Factors 2019 Jordan. Amman: The Jordanian Ministry of Health (2019). Available online at: www.who.int

