# Supplementary figures and images for: The development of national growth charts for Jordanian children aged 0–2 years
Source: Front Pediatr. 2025 Aug 12;13:1547581. doi: 10.3389/fped.2025.1547581 (PMC12378259; doi:10.3389/fped.2025.1547581)

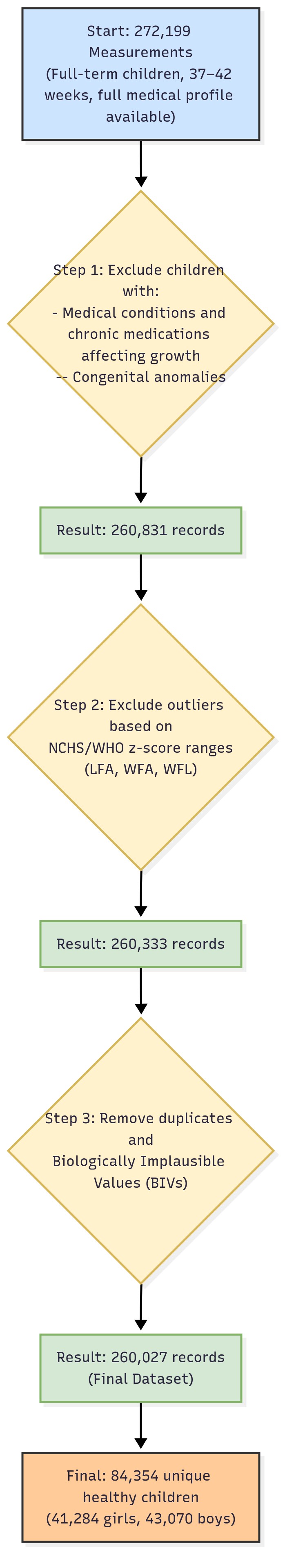

Supplement: Supplementary Figure A1 — Flow chart of the sample pre-processing and cleaning. [file Image1.jpeg]
