# Supplementary material for: The development of national growth charts for Jordanian children aged 0–2 years
Source: Front Pediatr. 2025 Aug 12;13:1547581. doi: 10.3389/fped.2025.1547581 (PMC12378259; doi:10.3389/fped.2025.1547581)
Supplement: Supplementary file 2 [file Datasheet1.docx]

Supplementary Material

1. **Supplementary tables**

S1. Sample distribution by governate/region

| Governorate/Region | Final sample N (%) | | | Original sample N (%) | | | Population (%) |
| --- | --- | --- | --- | --- | --- | --- | --- |
|  | Boys | Girls | Total | Boys | Girls | Total |  |
| **Northern Region** | 39,046 (29.49%) | 35,929 (28.16%) | 74,975 (28.83%) | 40,074 (28.83%) | 38,392 (28.83%) | 78,466 (28.83%) | 29 |
| Irbid | 25,504 (19.26%) | 24,142 (18.92%) | 49,646 (19.09%) | 26,532 (19.09%) | 25,418 (19.09%) | 51,950 (19.09%) | 19.2 |
| Ajloun | 2,211 (1.67%) | 1,924 (1.51%) | 4,135 (1.59%) | 2,211 (1.59%) | 2,118 (1.59%) | 4,329 (1.59%) | 1.6 |
| Jerash | 3,869 (2.92%) | 3,360 (2.63%) | 7,229 (2.78%) | 3,869 (2.78%) | 3,707 (2.78%) | 7,576 (2.78%) | 2.8 |
| Mafraq | 7,462 (5.63%) | 6,503 (5.10%) | 13,965 (5.37%) | 7,462 (5.37%) | 7,149 (5.37%) | 14,611 (5.37%) | 5.4 |
| **Central Region** | 82,598 (62.37%) | 82,359 (64.54%) | 164,957 (63.44%) | 88,166 (63.42%) | 84,463 (63.42%) | 172,629 (63.42%) | 63.8 |
| Amman | 53,651 (40.51%) | 54,931 (43.05%) | 108,582 (41.76%) | 58,040 (41.75%) | 55,602 (41.75%) | 113,642 (41.75%) | 42 |
| Balqa | 7,672 (5.79%) | 6,813 (5.34%) | 14,485 (5.57%) | 7,739 (5.57%) | 7,414 (5.57%) | 15,153 (5.57%) | 5.6 |
| Zarqa | 18,511 (13.98%) | 18,204 (14.27%) | 36,715 (14.12%) | 19,623 (14.12%) | 18,799 (14.12%) | 38,422 (14.12%) | 14.2 |
| Madaba | 2,764 (2.09%) | 2,411 (1.89%) | 5,175 (1.99%) | 2,764 (1.99%) | 2,648 (1.99%) | 5,412 (1.99%) | 2 |
| **Southern Region** | 10,779 (8.14%) | 9,376 (7.35%) | 20,155 (7.75%) | 10,779 (7.75%) | 10,325 (7.75%) | 21,104 (7.75%) | 7.8 |
| Karak | 4,146 (3.13%) | 3,604 (2.82%) | 7,750 (2.98%) | 4,146 (2.98%) | 3,971 (2.98%) | 8,117 (2.98%) | 3 |
| Tafila | 1,520 (1.15%) | 1,315 (1.03%) | 2,835 (1.09%) | 1,520 (1.09%) | 1,456 (1.09%) | 2,976 (1.09%) | 1.1 |
| Maan | 2,626 (1.98%) | 2,289 (1.79%) | 4,915 (1.89%) | 2,626 (1.89%) | 2,515 (1.89%) | 5,141 (1.89%) | 1.9 |
| Aqaba | 2,487 (1.88%) | 2,168 (1.70%) | 4,655 (1.79%) | 2,487 (1.79%) | 2,383 (1.79%) | 4,870 (1.79%) | 1.8 |
| Total | 132,423 (100%) | 127,604 (100%) | 260,027 (100%) | 139,019 (100%) | 133,180 (100%) | 272,199 (100%) | 100 |

Table S2. Frequency of Follow-up Measurements by Sex

| Number of Follow-up Visits | Children (Boys) | Children (Girls) | Total Children |
| --- | --- | --- | --- |
| 0 (1 Measurement) | 5075 | 4714 | 9789 |
| 1 | 13764 | 13224 | 26988 |
| 2 | 9024 | 8486 | 17510 |
| 3 | 5937 | 5768 | 11705 |
| 4 | 4138 | 4050 | 8188 |
| 5 | 2744 | 2727 | 5471 |
| 6 | 1412 | 1328 | 2740 |
| 7 | 190 | 207 | 397 |
| 8 | 38 | 32 | 70 |
| 9 (10 Measurements) | 10 | 6 | 16 |

Table S3. Centiles and equation parameters for length-for-age and weight-for-age in 0–24-month-old Jordanian toddlers

| Age (Day) | Nu | M | S | Tau | C1 | C3 | C5 | C10 | C15 | C25 | C50 | C75 | C85 | C90 | C95 | C97 | C99 |
| --- | --- | --- | --- | --- | --- | --- | --- | --- | --- | --- | --- | --- | --- | --- | --- | --- | --- |
| Length-for-Age (boys) | | | | | | | | | | | | | | | | | |
| 0 | 53.852 | 49.007 | -3.945 | -8.873 | 44.55 | 45.26 | 45.66 | 46.31 | 46.77 | 47.5 | 49 | 50.45 | 51.12 | 51.52 | 52.08 | 52.41 | 52.99 |
| 7 | -1.577 | 50.016 | -3.373 | 1.088 | 46.6 | 47.04 | 47.3 | 47.75 | 48.09 | 48.67 | 50.01 | 51.52 | 52.29 | 52.79 | 53.5 | 53.95 | 54.78 |
| 14 | 0.569 | 50.87 | -3.29 | 1.161 | 46.9 | 47.43 | 47.75 | 48.28 | 48.68 | 49.35 | 50.87 | 52.46 | 53.22 | 53.69 | 54.34 | 54.73 | 55.42 |
| 21 | -0.459 | 51.74 | -3.261 | 1.383 | 47.9 | 48.34 | 48.61 | 49.09 | 49.47 | 50.13 | 51.74 | 53.47 | 54.28 | 54.76 | 55.41 | 55.79 | 56.44 |
| 30 | -1.547 | 52.475 | -3.225 | 1.529 | 48.69 | 49.08 | 49.33 | 49.77 | 50.14 | 50.8 | 52.47 | 54.35 | 55.22 | 55.74 | 56.42 | 56.82 | 57.5 |
| 60 | 1.871 | 57.599 | -3.244 | 1.44 | 52.91 | 53.5 | 53.86 | 54.47 | 54.94 | 55.75 | 57.59 | 59.41 | 60.2 | 60.65 | 61.23 | 61.56 | 62.11 |
| 90 | 1.008 | 60.443 | -3.295 | 1.282 | 55.82 | 56.44 | 56.81 | 57.43 | 57.9 | 58.68 | 60.44 | 62.21 | 63.01 | 63.48 | 64.12 | 64.5 | 65.14 |
| 120 | 0.309 | 62.862 | -3.292 | 1.238 | 58.13 | 58.78 | 59.16 | 59.79 | 60.27 | 61.07 | 62.86 | 64.69 | 65.53 | 66.04 | 66.74 | 67.15 | 67.87 |
| 150 | 1.021 | 64.883 | -3.261 | 1.257 | 59.74 | 60.46 | 60.87 | 61.57 | 62.09 | 62.96 | 64.88 | 66.79 | 67.66 | 68.18 | 68.87 | 69.28 | 69.99 |
| 180 | 1.455 | 66.973 | -3.279 | 1.3 | 61.71 | 62.44 | 62.87 | 63.58 | 64.12 | 65.01 | 66.97 | 68.89 | 69.74 | 70.25 | 70.92 | 71.31 | 71.98 |
| 210 | 0.953 | 68.479 | -3.299 | 1.329 | 63.35 | 64.04 | 64.44 | 65.13 | 65.65 | 66.52 | 68.47 | 70.41 | 71.27 | 71.78 | 72.46 | 72.85 | 73.53 |
| 240 | 0.421 | 69.917 | -3.308 | 1.347 | 64.84 | 65.51 | 65.91 | 66.58 | 67.09 | 67.95 | 69.91 | 71.88 | 72.76 | 73.29 | 73.98 | 74.39 | 75.08 |
| 270 | 0.288 | 71.626 | -3.306 | 1.352 | 66.46 | 67.14 | 67.54 | 68.22 | 68.74 | 69.62 | 71.62 | 73.64 | 74.54 | 75.07 | 75.78 | 76.2 | 76.91 |
| 300 | 0.544 | 72.913 | -3.3 | 1.341 | 67.56 | 68.28 | 68.7 | 69.41 | 69.95 | 70.86 | 72.91 | 74.95 | 75.86 | 76.4 | 77.12 | 77.54 | 78.25 |
| 330 | 0.851 | 74.128 | -3.293 | 1.322 | 68.57 | 69.33 | 69.78 | 70.53 | 71.09 | 72.03 | 74.12 | 76.19 | 77.11 | 77.66 | 78.38 | 78.81 | 79.53 |
| 360 | 1.054 | 75.272 | -3.287 | 1.299 | 69.53 | 70.33 | 70.8 | 71.58 | 72.17 | 73.14 | 75.27 | 77.36 | 78.29 | 78.84 | 79.58 | 80.01 | 80.75 |
| 390 | 1.037 | 76.614 | -3.284 | 1.272 | 70.73 | 71.57 | 72.05 | 72.86 | 73.46 | 74.45 | 76.61 | 78.73 | 79.67 | 80.24 | 81 | 81.44 | 82.21 |
| 420 | 0.834 | 77.625 | -3.285 | 1.256 | 71.7 | 72.55 | 73.04 | 73.85 | 74.46 | 75.45 | 77.62 | 79.76 | 80.72 | 81.3 | 82.07 | 82.53 | 83.33 |
| 450 | 0.544 | 78.587 | -3.288 | 1.245 | 72.67 | 73.51 | 74 | 74.82 | 75.42 | 76.41 | 78.58 | 80.74 | 81.71 | 82.31 | 83.1 | 83.58 | 84.4 |
| 480 | 0.137 | 79.728 | -3.289 | 1.24 | 73.82 | 74.66 | 75.14 | 75.95 | 76.55 | 77.54 | 79.72 | 81.91 | 82.91 | 83.52 | 84.34 | 84.83 | 85.68 |
| 510 | -0.178 | 80.597 | -3.285 | 1.244 | 74.68 | 75.51 | 75.99 | 76.8 | 77.4 | 78.39 | 80.59 | 82.82 | 83.84 | 84.46 | 85.3 | 85.81 | 86.69 |
| 540 | -0.457 | 81.43 | -3.276 | 1.255 | 75.48 | 76.3 | 76.78 | 77.58 | 78.19 | 79.19 | 81.43 | 83.7 | 84.75 | 85.39 | 86.25 | 86.77 | 87.67 |
| 570 | -0.688 | 82.223 | -3.261 | 1.273 | 76.19 | 77.02 | 77.49 | 78.3 | 78.92 | 79.93 | 82.22 | 84.56 | 85.64 | 86.3 | 87.19 | 87.72 | 88.64 |
| 600 | -0.93 | 83.156 | -3.233 | 1.305 | 76.99 | 77.81 | 78.29 | 79.11 | 79.73 | 80.77 | 83.15 | 85.6 | 86.73 | 87.42 | 88.34 | 88.89 | 89.84 |
| 630 | -1.104 | 83.855 | -3.205 | 1.339 | 77.54 | 78.36 | 78.85 | 79.68 | 80.31 | 81.38 | 83.85 | 86.41 | 87.58 | 88.29 | 89.24 | 89.81 | 90.79 |
| 660 | -1.272 | 84.511 | -3.173 | 1.379 | 78.04 | 78.86 | 79.34 | 80.18 | 80.83 | 81.93 | 84.51 | 87.19 | 88.41 | 89.15 | 90.13 | 90.71 | 91.71 |
| 690 | -1.487 | 85.27 | -3.129 | 1.438 | 78.59 | 79.4 | 79.88 | 80.74 | 81.4 | 82.55 | 85.27 | 88.12 | 89.42 | 90.2 | 91.22 | 91.82 | 92.85 |
| 720 | -1.719 | 85.964 | -3.082 | 1.506 | 79.06 | 79.86 | 80.35 | 81.21 | 81.88 | 83.08 | 85.96 | 89.02 | 90.4 | 91.22 | 92.29 | 92.91 | 93.97 |
| Weight-for-age (boys) | | | | | | | | | | | | | | | | | |
| 0 | 0.12 | 3.063 | -2.017 | 1.114 | 2.29 | 2.4 | 2.46 | 2.56 | 2.64 | 2.77 | 3.06 | 3.38 | 3.54 | 3.64 | 3.78 | 3.87 | 4.04 |
| 7 | 0.332 | 3.496 | -2.103 | 1.107 | 2.66 | 2.78 | 2.84 | 2.96 | 3.04 | 3.18 | 3.49 | 3.82 | 3.98 | 4.08 | 4.23 | 4.32 | 4.48 |
| 14 | 0.694 | 3.707 | -2.096 | 1.105 | 2.77 | 2.91 | 2.99 | 3.12 | 3.21 | 3.37 | 3.7 | 4.05 | 4.21 | 4.32 | 4.46 | 4.55 | 4.71 |
| 21 | 0.977 | 3.952 | -2.079 | 1.104 | 2.9 | 3.06 | 3.15 | 3.3 | 3.41 | 3.58 | 3.95 | 4.32 | 4.49 | 4.6 | 4.75 | 4.84 | 5 |
| 30 | 1.147 | 4.219 | -2.092 | 1.103 | 3.08 | 3.26 | 3.36 | 3.52 | 3.64 | 3.82 | 4.21 | 4.6 | 4.78 | 4.89 | 5.05 | 5.14 | 5.3 |
| 60 | 1.011 | 5.474 | -2.22 | 1.1 | 4.2 | 4.4 | 4.51 | 4.68 | 4.82 | 5.02 | 5.47 | 5.91 | 6.12 | 6.25 | 6.43 | 6.54 | 6.73 |
| 90 | 0.858 | 6.215 | -2.269 | 1.099 | 4.87 | 5.07 | 5.18 | 5.37 | 5.51 | 5.73 | 6.21 | 6.69 | 6.92 | 7.07 | 7.26 | 7.38 | 7.6 |
| 120 | 0.821 | 6.814 | -2.289 | 1.097 | 5.37 | 5.59 | 5.71 | 5.91 | 6.06 | 6.3 | 6.81 | 7.33 | 7.58 | 7.73 | 7.94 | 8.07 | 8.31 |
| 150 | 0.772 | 7.344 | -2.278 | 1.096 | 5.78 | 6.01 | 6.14 | 6.36 | 6.52 | 6.78 | 7.34 | 7.91 | 8.18 | 8.35 | 8.58 | 8.72 | 8.98 |
| 180 | 0.724 | 7.85 | -2.256 | 1.095 | 6.15 | 6.4 | 6.55 | 6.78 | 6.96 | 7.24 | 7.85 | 8.47 | 8.76 | 8.95 | 9.21 | 9.37 | 9.65 |
| 210 | 0.723 | 8.225 | -2.255 | 1.094 | 6.44 | 6.71 | 6.86 | 7.11 | 7.29 | 7.58 | 8.22 | 8.87 | 9.18 | 9.38 | 9.65 | 9.82 | 10.11 |
| 240 | 0.737 | 8.639 | -2.274 | 1.094 | 6.8 | 7.07 | 7.23 | 7.48 | 7.67 | 7.98 | 8.63 | 9.31 | 9.63 | 9.83 | 10.11 | 10.28 | 10.58 |
| 270 | 0.735 | 9.139 | -2.308 | 1.093 | 7.26 | 7.54 | 7.69 | 7.96 | 8.15 | 8.46 | 9.13 | 9.82 | 10.15 | 10.35 | 10.64 | 10.81 | 11.12 |
| 300 | 0.699 | 9.468 | -2.329 | 1.092 | 7.56 | 7.84 | 8.01 | 8.27 | 8.47 | 8.78 | 9.46 | 10.16 | 10.49 | 10.7 | 10.99 | 11.17 | 11.49 |
| 330 | 0.638 | 9.736 | -2.34 | 1.092 | 7.81 | 8.09 | 8.26 | 8.52 | 8.72 | 9.04 | 9.73 | 10.44 | 10.78 | 11 | 11.29 | 11.48 | 11.8 |
| 360 | 0.569 | 9.976 | -2.343 | 1.091 | 8.02 | 8.31 | 8.47 | 8.74 | 8.94 | 9.27 | 9.97 | 10.7 | 11.05 | 11.27 | 11.58 | 11.76 | 12.1 |
| 390 | 0.493 | 10.271 | -2.338 | 1.09 | 8.27 | 8.56 | 8.72 | 9 | 9.21 | 9.54 | 10.27 | 11.02 | 11.39 | 11.62 | 11.94 | 12.13 | 12.49 |
| 420 | 0.451 | 10.51 | -2.331 | 1.09 | 8.45 | 8.75 | 8.92 | 9.21 | 9.42 | 9.76 | 10.51 | 11.28 | 11.66 | 11.9 | 12.23 | 12.44 | 12.8 |
| 450 | 0.426 | 10.743 | -2.328 | 1.089 | 8.64 | 8.94 | 9.12 | 9.41 | 9.62 | 9.97 | 10.74 | 11.54 | 11.92 | 12.17 | 12.51 | 12.72 | 13.1 |
| 480 | 0.412 | 11.019 | -2.333 | 1.089 | 8.88 | 9.19 | 9.36 | 9.66 | 9.88 | 10.23 | 11.01 | 11.83 | 12.23 | 12.48 | 12.83 | 13.04 | 13.43 |
| 510 | 0.41 | 11.225 | -2.347 | 1.088 | 9.07 | 9.38 | 9.56 | 9.86 | 10.08 | 10.44 | 11.22 | 12.04 | 12.44 | 12.69 | 13.04 | 13.26 | 13.65 |
| 540 | 0.413 | 11.414 | -2.363 | 1.088 | 9.25 | 9.57 | 9.75 | 10.04 | 10.26 | 10.62 | 11.41 | 12.23 | 12.63 | 12.88 | 13.23 | 13.45 | 13.84 |
| 570 | 0.417 | 11.587 | -2.375 | 1.088 | 9.42 | 9.73 | 9.91 | 10.21 | 10.43 | 10.79 | 11.58 | 12.4 | 12.8 | 13.05 | 13.41 | 13.62 | 14.01 |
| 600 | 0.424 | 11.781 | -2.379 | 1.087 | 9.58 | 9.9 | 10.08 | 10.39 | 10.61 | 10.98 | 11.78 | 12.61 | 13.01 | 13.27 | 13.62 | 13.84 | 14.24 |
| 630 | 0.428 | 11.917 | -2.368 | 1.087 | 9.67 | 10 | 10.18 | 10.49 | 10.72 | 11.1 | 11.91 | 12.76 | 13.17 | 13.44 | 13.8 | 14.02 | 14.43 |
| 660 | 0.43 | 12.043 | -2.347 | 1.086 | 9.72 | 10.06 | 10.25 | 10.57 | 10.81 | 11.2 | 12.04 | 12.92 | 13.34 | 13.61 | 13.99 | 14.22 | 14.64 |
| 690 | 0.43 | 12.19 | -2.308 | 1.086 | 9.76 | 10.11 | 10.31 | 10.65 | 10.9 | 11.3 | 12.19 | 13.11 | 13.56 | 13.84 | 14.24 | 14.48 | 14.93 |
| 720 | 0.427 | 12.335 | -2.26 | 1.086 | 9.76 | 10.13 | 10.35 | 10.7 | 10.96 | 11.39 | 12.33 | 13.31 | 13.79 | 14.09 | 14.52 | 14.78 | 15.25 |
| Length-for-age (girls) | | | | | | | | | | | | | | | | | |
| 0 | -3.529 | 49.101 | 2.83 | -2.445 | 45.73 | 46.12 | 46.35 | 46.78 | 47.11 | 47.69 | 49.03 | 50.32 | 50.85 | 51.14 | 51.51 | 52.03 | 52.03 |
| 7 | 8.891 | 49.621 | -3.332 | 1.614 | 45.61 | 46.27 | 46.64 | 47.23 | 47.64 | 48.29 | 49.62 | 51 | 51.72 | 52.21 | 52.91 | 54.21 | 54.21 |
| 14 | 15.903 | 50.114 | -3.287 | 1.624 | 45.86 | 46.53 | 46.91 | 47.53 | 47.97 | 48.67 | 50.14 | 51.61 | 52.31 | 52.75 | 53.37 | 54.43 | 54.43 |
| 21 | 17.273 | 51.005 | -3.28 | 1.651 | 47.12 | 47.66 | 47.98 | 48.5 | 48.9 | 49.56 | 51.04 | 52.62 | 53.38 | 53.86 | 54.53 | 55.66 | 55.66 |
| 30 | 19.21 | 51.655 | -3.21 | 1.835 | 47.74 | 48.2 | 48.48 | 48.98 | 49.37 | 50.05 | 51.7 | 53.5 | 54.33 | 54.83 | 55.5 | 56.57 | 56.57 |
| 60 | 28.88 | 56.446 | -3.229 | 1.551 | 51.8 | 52.44 | 52.81 | 53.44 | 53.92 | 54.73 | 56.53 | 58.31 | 59.09 | 59.55 | 60.16 | 61.12 | 61.12 |
| 90 | 32.699 | 58.949 | -3.278 | 1.299 | 54.13 | 54.87 | 55.29 | 55.98 | 56.49 | 57.3 | 59.04 | 60.74 | 61.5 | 61.97 | 62.59 | 63.6 | 63.6 |
| 120 | 34.249 | 61.297 | -3.289 | 1.253 | 56.59 | 57.28 | 57.68 | 58.34 | 58.83 | 59.64 | 61.4 | 63.16 | 63.96 | 64.45 | 65.11 | 66.18 | 66.18 |
| 150 | 35.955 | 63.213 | -3.285 | 1.249 | 58.46 | 59.13 | 59.52 | 60.18 | 60.68 | 61.5 | 63.33 | 65.16 | 65.99 | 66.49 | 67.16 | 68.23 | 68.23 |
| 180 | 38.677 | 65.091 | -3.28 | 1.225 | 60.08 | 60.79 | 61.21 | 61.91 | 62.43 | 63.3 | 65.21 | 67.09 | 67.92 | 68.41 | 69.06 | 70.09 | 70.09 |
| 210 | 40.42 | 66.52 | -3.278 | 1.18 | 61.31 | 62.07 | 62.51 | 63.24 | 63.79 | 64.69 | 66.65 | 68.55 | 69.38 | 69.88 | 70.53 | 71.56 | 71.56 |
| 240 | 41.484 | 68.013 | -3.276 | 1.128 | 62.7 | 63.48 | 63.93 | 64.68 | 65.24 | 66.15 | 68.15 | 70.09 | 70.94 | 71.45 | 72.12 | 73.19 | 73.19 |
| 270 | 42.282 | 69.881 | -3.273 | 1.064 | 64.52 | 65.3 | 65.75 | 66.51 | 67.07 | 68 | 70.02 | 72.02 | 72.91 | 73.44 | 74.16 | 75.31 | 75.31 |
| 300 | 42.874 | 71.287 | -3.27 | 1.017 | 65.86 | 66.66 | 67.11 | 67.88 | 68.45 | 69.38 | 71.43 | 73.47 | 74.39 | 74.95 | 75.7 | 76.92 | 76.92 |
| 330 | 43.603 | 72.575 | -3.265 | 0.978 | 67.05 | 67.86 | 68.33 | 69.11 | 69.69 | 70.64 | 72.72 | 74.8 | 75.75 | 76.32 | 77.1 | 78.36 | 78.36 |
| 360 | 44.537 | 73.736 | -3.261 | 0.945 | 68.06 | 68.91 | 69.39 | 70.19 | 70.79 | 71.77 | 73.89 | 76 | 76.95 | 77.54 | 78.32 | 79.6 | 79.6 |
| 390 | 45.812 | 75.051 | -3.257 | 0.915 | 69.17 | 70.06 | 70.56 | 71.41 | 72.03 | 73.03 | 75.21 | 77.34 | 78.31 | 78.89 | 79.68 | 80.95 | 80.95 |
| 420 | 46.756 | 76.037 | -3.255 | 0.898 | 70.01 | 70.93 | 71.45 | 72.32 | 72.95 | 73.98 | 76.2 | 78.35 | 79.32 | 79.9 | 80.68 | 81.95 | 81.95 |
| 450 | 47.541 | 76.99 | -3.255 | 0.883 | 70.86 | 71.8 | 72.33 | 73.21 | 73.86 | 74.91 | 77.15 | 79.33 | 80.3 | 80.88 | 81.66 | 82.93 | 82.93 |
| 480 | 48.219 | 78.156 | -3.258 | 0.865 | 71.99 | 72.93 | 73.46 | 74.35 | 75 | 76.06 | 78.32 | 80.52 | 81.5 | 82.1 | 82.89 | 84.16 | 84.16 |
| 510 | 48.482 | 79.085 | -3.262 | 0.85 | 72.95 | 73.88 | 74.4 | 75.28 | 75.93 | 76.98 | 79.25 | 81.48 | 82.47 | 83.08 | 83.89 | 85.19 | 85.19 |
| 540 | 48.516 | 80.008 | -3.263 | 0.834 | 73.95 | 74.84 | 75.36 | 76.22 | 76.86 | 77.9 | 80.18 | 82.44 | 83.46 | 84.08 | 84.92 | 86.28 | 86.28 |
| 570 | 48.472 | 80.896 | -3.26 | 0.815 | 74.88 | 75.76 | 76.27 | 77.11 | 77.75 | 78.78 | 81.07 | 83.37 | 84.43 | 85.07 | 85.94 | 87.37 | 87.37 |
| 600 | 48.523 | 81.912 | -3.248 | 0.788 | 75.88 | 76.75 | 77.25 | 78.1 | 78.73 | 79.77 | 82.09 | 84.46 | 85.56 | 86.24 | 87.16 | 88.69 | 88.69 |
| 630 | 48.773 | 82.625 | -3.23 | 0.763 | 76.47 | 77.36 | 77.87 | 78.73 | 79.38 | 80.44 | 82.8 | 85.23 | 86.37 | 87.08 | 88.04 | 89.64 | 89.64 |
| 660 | 49.293 | 83.233 | -3.204 | 0.735 | 76.84 | 77.77 | 78.31 | 79.21 | 79.88 | 80.98 | 83.41 | 85.91 | 87.09 | 87.81 | 88.81 | 90.64 | 90.47 |
| 690 | 50.404 | 83.832 | -3.161 | 0.695 | 76.91 | 77.95 | 78.54 | 79.53 | 80.26 | 81.44 | 84.01 | 86.61 | 87.81 | 88.56 | 89.58 | 91.29 | 91.29 |
| 720 | 52.093 | 84.24 | -3.103 | 0.65 | 76.38 | 77.64 | 78.35 | 79.5 | 80.33 | 81.66 | 84.42 | 87.11 | 88.34 | 89.1 | 90.12 | 91.81 | 91.81 |
| Weight-for-age (girls) | | | | | | | | | | | | | | | | | |
| 0 | 0.164 | 2.9254 | -1.673 | 5.92 | 2.1 | 2.13 | 2.16 | 2.24 | 2.32 | 2.48 | 2.92 | 3.43 | 3.65 | 3.77 | 3.89 | 3.94 | 3.98 |
| 7 | 0.329 | 3.403 | -2.139 | 1.275 | 2.64 | 2.74 | 2.79 | 2.89 | 2.97 | 3.1 | 3.4 | 3.72 | 3.87 | 3.96 | 4.08 | 4.16 | 4.29 |
| 14 | 0.896 | 3.566 | -2.135 | 1.265 | 2.71 | 2.82 | 2.89 | 3.01 | 3.09 | 3.24 | 3.56 | 3.89 | 4.04 | 4.13 | 4.25 | 4.32 | 4.44 |
| 21 | 1.181 | 3.793 | -2.138 | 1.161 | 2.83 | 2.97 | 3.05 | 3.19 | 3.29 | 3.45 | 3.79 | 4.12 | 4.28 | 4.37 | 4.5 | 4.58 | 4.71 |
| 30 | 1.298 | 4.001 | -2.109 | 1.059 | 2.91 | 3.09 | 3.18 | 3.34 | 3.45 | 3.63 | 4 | 4.35 | 4.52 | 4.62 | 4.76 | 4.85 | 5 |
| 60 | 1.04 | 5.1 | -2.229 | 1.038 | 3.91 | 4.1 | 4.2 | 4.37 | 4.49 | 4.69 | 5.1 | 5.5 | 5.69 | 5.82 | 5.98 | 6.09 | 6.27 |
| 90 | 0.783 | 5.705 | -2.266 | 1.026 | 4.46 | 4.65 | 4.76 | 4.93 | 5.06 | 5.27 | 5.7 | 6.14 | 6.35 | 6.49 | 6.68 | 6.8 | 7.01 |
| 120 | 0.63 | 6.307 | -2.295 | 1.082 | 5 | 5.19 | 5.3 | 5.49 | 5.62 | 5.84 | 6.3 | 6.78 | 7.01 | 7.16 | 7.36 | 7.49 | 7.71 |
| 150 | 0.552 | 6.792 | -2.291 | 1.061 | 5.39 | 5.6 | 5.71 | 5.91 | 6.05 | 6.29 | 6.79 | 7.31 | 7.56 | 7.72 | 7.94 | 8.08 | 8.33 |
| 180 | 0.528 | 7.24 | -2.255 | 1.023 | 5.68 | 5.92 | 6.05 | 6.27 | 6.43 | 6.69 | 7.24 | 7.8 | 8.09 | 8.27 | 8.52 | 8.68 | 8.96 |
| 210 | 0.544 | 7.623 | -2.236 | 1.056 | 5.96 | 6.21 | 6.35 | 6.58 | 6.75 | 7.03 | 7.62 | 8.23 | 8.53 | 8.72 | 8.99 | 9.16 | 9.46 |
| 240 | 0.573 | 8.049 | -2.237 | 1.145 | 6.32 | 6.57 | 6.71 | 6.94 | 7.12 | 7.41 | 8.04 | 8.7 | 9.02 | 9.21 | 9.48 | 9.65 | 9.94 |
| 270 | 0.61 | 8.531 | -2.254 | 1.23 | 6.75 | 6.99 | 7.13 | 7.37 | 7.55 | 7.85 | 8.53 | 9.22 | 9.55 | 9.75 | 10.02 | 10.18 | 10.46 |
| 300 | 0.635 | 8.848 | -2.271 | 1.226 | 7.03 | 7.27 | 7.42 | 7.66 | 7.85 | 8.15 | 8.84 | 9.55 | 9.88 | 10.08 | 10.36 | 10.52 | 10.81 |
| 330 | 0.653 | 9.126 | -2.286 | 1.168 | 7.25 | 7.51 | 7.67 | 7.92 | 8.11 | 8.43 | 9.12 | 9.83 | 10.17 | 10.38 | 10.66 | 10.83 | 11.14 |
| 360 | 0.663 | 9.381 | -2.296 | 1.098 | 7.44 | 7.73 | 7.89 | 8.16 | 8.36 | 8.68 | 9.38 | 10.09 | 10.43 | 10.65 | 10.95 | 11.13 | 11.45 |
| 390 | 0.664 | 9.682 | -2.304 | 1.034 | 7.67 | 7.98 | 8.15 | 8.44 | 8.64 | 8.97 | 9.68 | 10.4 | 10.75 | 10.98 | 11.29 | 11.48 | 11.83 |
| 420 | 0.657 | 9.914 | -2.307 | 1.022 | 7.86 | 8.17 | 8.35 | 8.64 | 8.85 | 9.19 | 9.91 | 10.64 | 11 | 11.23 | 11.56 | 11.76 | 12.12 |
| 450 | 0.646 | 10.142 | -2.31 | 1.039 | 8.05 | 8.37 | 8.55 | 8.84 | 9.06 | 9.4 | 10.14 | 10.89 | 11.26 | 11.49 | 11.82 | 12.02 | 12.38 |
| 480 | 0.628 | 10.423 | -2.312 | 1.081 | 8.3 | 8.62 | 8.8 | 9.09 | 9.31 | 9.66 | 10.42 | 11.2 | 11.57 | 11.81 | 12.14 | 12.34 | 12.71 |
| 510 | 0.612 | 10.639 | -2.314 | 1.119 | 8.5 | 8.81 | 8.99 | 9.28 | 9.5 | 9.86 | 10.63 | 11.44 | 11.82 | 12.06 | 12.39 | 12.59 | 12.95 |
| 540 | 0.596 | 10.843 | -2.317 | 1.152 | 8.69 | 8.99 | 9.17 | 9.46 | 9.68 | 10.04 | 10.84 | 11.66 | 12.05 | 12.29 | 12.62 | 12.82 | 13.18 |
| 570 | 0.579 | 11.03 | -2.319 | 1.175 | 8.85 | 9.16 | 9.33 | 9.63 | 9.85 | 10.22 | 11.03 | 11.86 | 12.26 | 12.5 | 12.84 | 13.04 | 13.4 |
| 600 | 0.559 | 11.239 | -2.321 | 1.188 | 9.04 | 9.34 | 9.52 | 9.82 | 10.04 | 10.41 | 11.23 | 12.09 | 12.49 | 12.74 | 13.08 | 13.28 | 13.64 |
| 630 | 0.544 | 11.385 | -2.322 | 1.188 | 9.16 | 9.47 | 9.65 | 9.95 | 10.17 | 10.55 | 11.38 | 12.24 | 12.65 | 12.9 | 13.25 | 13.45 | 13.82 |
| 660 | 0.53 | 11.516 | -2.322 | 1.181 | 9.26 | 9.58 | 9.76 | 10.06 | 10.29 | 10.67 | 11.51 | 12.38 | 12.8 | 13.05 | 13.4 | 13.61 | 13.99 |
| 690 | 0.514 | 11.662 | -2.321 | 1.164 | 9.38 | 9.7 | 9.88 | 10.19 | 10.42 | 10.81 | 11.66 | 12.54 | 12.96 | 13.22 | 13.58 | 13.79 | 14.18 |
| 720 | 0.5 | 11.791 | -2.318 | 1.142 | 9.47 | 9.79 | 9.98 | 10.3 | 10.54 | 10.93 | 11.79 | 12.68 | 13.1 | 13.37 | 13.74 | 13.96 | 14.36 |

Table S4. Standard deviation (SD) for length-for-age and weight-for-age in 0–24-month-old Jordanian toddlers

| Days | -3 SD | -2 SD | -1 SD | 1 SD | 2 SD | 3 SD |
| --- | --- | --- | --- | --- | --- | --- |
| Length-for-Age (boys) | | | | | | |
| 0 | 43.55 | 45.07 | 46.84 | 51.06 | 52.57 | 53.79 |
| 7 | 46.02 | 46.92 | 48.15 | 52.22 | 54.18 | 56.01 |
| 14 | 46.20 | 47.29 | 48.74 | 53.15 | 54.93 | 56.38 |
| 30 | 47.34 | 48.22 | 49.54 | 54.21 | 55.97 | 57.32 |
| 60 | 50.73 | 51.69 | 53.17 | 58.24 | 59.86 | 60.96 |
| 90 | 53.55 | 54.83 | 56.56 | 61.62 | 63.28 | 64.49 |
| 120 | 56.27 | 57.55 | 59.25 | 64.22 | 66.05 | 67.48 |
| 150 | 58.08 | 59.48 | 61.31 | 66.58 | 68.51 | 70.01 |
| 180 | 59.52 | 61.04 | 63.01 | 68.46 | 70.33 | 71.74 |
| 210 | 61.17 | 62.65 | 64.60 | 70.06 | 71.89 | 73.25 |
| 240 | 62.84 | 64.23 | 66.11 | 71.57 | 73.43 | 74.81 |
| 270 | 64.32 | 65.68 | 67.52 | 73.05 | 74.96 | 76.38 |
| 300 | 65.57 | 66.95 | 68.83 | 74.46 | 76.40 | 77.85 |
| 330 | 66.63 | 68.08 | 70.04 | 75.78 | 77.74 | 79.20 |
| 360 | 67.56 | 69.12 | 71.19 | 77.03 | 79.01 | 80.49 |
| 390 | 68.46 | 70.12 | 72.27 | 78.21 | 80.22 | 81.73 |
| 420 | 69.36 | 71.09 | 73.31 | 79.32 | 81.38 | 82.94 |
| 450 | 70.32 | 72.08 | 74.31 | 80.38 | 82.49 | 84.11 |
| 480 | 71.28 | 73.04 | 75.28 | 81.38 | 83.55 | 85.23 |
| 510 | 72.24 | 73.98 | 76.21 | 82.35 | 84.57 | 86.31 |
| 540 | 73.14 | 74.86 | 77.08 | 83.29 | 85.57 | 87.36 |
| 570 | 73.98 | 75.69 | 77.90 | 84.20 | 86.54 | 88.40 |
| 600 | 74.75 | 76.44 | 78.66 | 85.10 | 87.50 | 89.41 |
| 630 | 75.44 | 77.12 | 79.35 | 85.98 | 88.45 | 90.40 |
| 660 | 76.06 | 77.73 | 79.98 | 86.84 | 89.39 | 91.38 |
| 690 | 76.61 | 78.27 | 80.55 | 87.68 | 90.31 | 92.34 |
| 720 | 77.11 | 78.75 | 81.06 | 88.50 | 91.22 | 93.29 |
| 750 | 77.56 | 79.17 | 81.51 | 89.30 | 92.12 | 94.22 |
| 780 | 77.97 | 79.55 | 81.91 | 90.08 | 93.00 | 95.13 |
| Weight-for-age (boys) | | | | | | |
| 0 | 2.05 | 2.28 | 2.59 | 3.54 | 3.97 | 4.35 |
| 7 | 2.51 | 2.75 | 3.06 | 3.97 | 4.37 | 4.71 |
| 14 | 2.60 | 2.88 | 3.23 | 4.20 | 4.60 | 4.93 |
| 30 | 2.69 | 3.02 | 3.43 | 4.48 | 4.89 | 5.23 |
| 60 | 3.51 | 3.91 | 4.39 | 5.58 | 6.04 | 6.40 |
| 90 | 4.30 | 4.71 | 5.22 | 6.55 | 7.07 | 7.50 |
| 120 | 4.85 | 5.28 | 5.82 | 7.23 | 7.79 | 8.26 |
| 150 | 5.29 | 5.76 | 6.33 | 7.87 | 8.48 | 9.00 |
| 180 | 5.62 | 6.12 | 6.74 | 8.41 | 9.08 | 9.64 |
| 210 | 5.88 | 6.41 | 7.07 | 8.84 | 9.56 | 10.17 |
| 240 | 6.17 | 6.72 | 7.41 | 9.27 | 10.02 | 10.65 |
| 270 | 6.52 | 7.10 | 7.81 | 9.71 | 10.48 | 11.12 |
| 300 | 6.88 | 7.46 | 8.19 | 10.12 | 10.90 | 11.56 |
| 330 | 7.18 | 7.77 | 8.50 | 10.47 | 11.26 | 11.93 |
| 360 | 7.43 | 8.02 | 8.76 | 10.75 | 11.57 | 12.26 |
| 390 | 7.64 | 8.23 | 8.98 | 11.02 | 11.86 | 12.58 |
| 420 | 7.83 | 8.43 | 9.19 | 11.29 | 12.16 | 12.91 |
| 450 | 8.01 | 8.63 | 9.40 | 11.56 | 12.47 | 13.25 |
| 480 | 8.19 | 8.82 | 9.61 | 11.83 | 12.76 | 13.57 |
| 510 | 8.37 | 9.01 | 9.82 | 12.08 | 13.03 | 13.86 |
| 540 | 8.56 | 9.20 | 10.02 | 12.30 | 13.27 | 14.10 |
| 570 | 8.74 | 9.39 | 10.21 | 12.50 | 13.47 | 14.30 |
| 600 | 8.92 | 9.57 | 10.39 | 12.68 | 13.65 | 14.48 |
| 630 | 9.07 | 9.72 | 10.55 | 12.85 | 13.82 | 14.66 |
| 660 | 9.18 | 9.84 | 10.68 | 13.02 | 14.00 | 14.85 |
| 690 | 9.25 | 9.93 | 10.79 | 13.18 | 14.19 | 15.06 |
| 720 | 9.28 | 9.98 | 10.87 | 13.35 | 14.39 | 15.29 |
| 750 | 9.29 | 10.02 | 10.94 | 13.52 | 14.61 | 15.55 |
| 780 | 9.27 | 10.03 | 11.00 | 13.70 | 14.85 | 15.84 |
| Length-for-Age (girls) | | | | | | |
| 0 | 45.24 | 46.01 | 47.17 | 50.81 | 51.80 | 52.43 |
| 7 | 44.68 | 46.10 | 47.71 | 51.66 | 53.59 | 55.48 |
| 14 | 44.92 | 46.35 | 48.04 | 52.25 | 53.94 | 55.39 |
| 30 | 46.41 | 47.52 | 48.97 | 53.31 | 55.14 | 56.70 |
| 60 | 49.57 | 50.63 | 52.19 | 57.39 | 59.09 | 60.28 |
| 90 | 51.99 | 53.48 | 55.35 | 60.24 | 61.92 | 63.20 |
| 120 | 54.42 | 55.92 | 57.79 | 62.69 | 64.44 | 65.81 |
| 150 | 56.72 | 58.11 | 59.91 | 64.98 | 66.81 | 68.23 |
| 180 | 58.25 | 59.65 | 61.50 | 66.73 | 68.54 | 69.91 |
| 210 | 59.41 | 60.91 | 62.85 | 68.21 | 69.99 | 71.30 |
| 240 | 60.61 | 62.20 | 64.23 | 69.69 | 71.48 | 72.81 |
| 270 | 62.02 | 63.64 | 65.70 | 71.26 | 73.13 | 74.52 |
| 300 | 63.46 | 65.09 | 67.17 | 72.83 | 74.79 | 76.28 |
| 330 | 64.80 | 66.44 | 68.54 | 74.31 | 76.37 | 77.95 |
| 360 | 65.96 | 67.64 | 69.78 | 75.66 | 77.79 | 79.44 |
| 390 | 66.93 | 68.68 | 70.89 | 76.87 | 79.02 | 80.70 |
| 420 | 67.77 | 69.60 | 71.89 | 77.96 | 80.11 | 81.78 |
| 450 | 68.56 | 70.47 | 72.83 | 78.98 | 81.13 | 82.78 |
| 480 | 69.37 | 71.33 | 73.74 | 79.97 | 82.11 | 83.75 |
| 510 | 70.24 | 72.21 | 74.65 | 80.93 | 83.09 | 84.74 |
| 540 | 71.19 | 73.14 | 75.57 | 81.90 | 84.09 | 85.76 |
| 570 | 72.22 | 74.11 | 76.50 | 82.88 | 85.13 | 86.86 |
| 600 | 73.24 | 75.07 | 77.41 | 83.85 | 86.19 | 88.02 |
| 630 | 74.15 | 75.94 | 78.26 | 84.80 | 87.24 | 89.18 |
| 660 | 74.88 | 76.68 | 79.01 | 85.67 | 88.23 | 90.29 |
| 690 | 75.39 | 77.24 | 79.62 | 86.46 | 89.12 | 91.29 |
| 720 | 75.62 | 77.58 | 80.09 | 87.14 | 89.89 | 92.13 |
| 750 | 75.50 | 77.67 | 80.38 | 87.70 | 90.51 | 92.78 |
| 780 | 74.88 | 77.42 | 80.47 | 88.14 | 90.95 | 93.21 |
| Weight-for-Age (girls) | | | | | | |
| 0 | 2.10 | 2.13 | 2.33 | 3.64 | 3.96 | 4.01 |
| 7 | 2.48 | 2.69 | 2.96 | 3.82 | 4.18 | 4.49 |
| 14 | 2.54 | 2.77 | 3.08 | 3.99 | 4.32 | 4.56 |
| 30 | 2.63 | 2.92 | 3.29 | 4.24 | 4.59 | 4.86 |
| 60 | 3.21 | 3.59 | 4.04 | 5.15 | 5.57 | 5.91 |
| 90 | 3.89 | 4.31 | 4.80 | 6.00 | 6.50 | 6.94 |
| 120 | 4.47 | 4.87 | 5.35 | 6.65 | 7.19 | 7.65 |
| 150 | 4.94 | 5.35 | 5.87 | 7.27 | 7.85 | 8.35 |
| 180 | 5.21 | 5.67 | 6.23 | 7.74 | 8.39 | 8.96 |
| 210 | 5.41 | 5.91 | 6.52 | 8.15 | 8.86 | 9.49 |
| 240 | 5.71 | 6.22 | 6.86 | 8.61 | 9.35 | 9.99 |
| 270 | 6.08 | 6.58 | 7.23 | 9.09 | 9.83 | 10.44 |
| 300 | 6.43 | 6.92 | 7.57 | 9.51 | 10.25 | 10.84 |
| 330 | 6.69 | 7.20 | 7.87 | 9.85 | 10.59 | 11.19 |
| 360 | 6.89 | 7.44 | 8.14 | 10.13 | 10.91 | 11.55 |
| 390 | 7.06 | 7.65 | 8.39 | 10.40 | 11.21 | 11.90 |
| 420 | 7.21 | 7.84 | 8.62 | 10.65 | 11.50 | 12.24 |
| 450 | 7.38 | 8.04 | 8.83 | 10.90 | 11.78 | 12.55 |
| 480 | 7.57 | 8.23 | 9.04 | 11.15 | 12.05 | 12.83 |
| 510 | 7.77 | 8.43 | 9.24 | 11.41 | 12.31 | 13.09 |
| 540 | 7.98 | 8.63 | 9.44 | 11.66 | 12.56 | 13.33 |
| 570 | 8.18 | 8.82 | 9.63 | 11.89 | 12.80 | 13.56 |
| 600 | 8.36 | 8.99 | 9.80 | 12.11 | 13.03 | 13.78 |
| 630 | 8.52 | 9.15 | 9.96 | 12.31 | 13.24 | 13.99 |
| 660 | 8.66 | 9.29 | 10.11 | 12.49 | 13.42 | 14.18 |
| 690 | 8.78 | 9.41 | 10.24 | 12.65 | 13.60 | 14.37 |
| 720 | 8.87 | 9.52 | 10.36 | 12.79 | 13.75 | 14.54 |
| 750 | 8.95 | 9.61 | 10.46 | 12.92 | 13.90 | 14.71 |
| 780 | 9.01 | 9.69 | 10.56 | 13.04 | 14.04 | 14.87 |

Table S5. Centiles and equation parameters for weight-for-length in 0–24-month-old Jordanian toddlers

| Length* (cm) | Nu | M | S | Tau | C1 | C3 | C5 | C10 | C15 | C25 | C50 | C75 | C85 | C90 | C95 | C97 | C99 |
| --- | --- | --- | --- | --- | --- | --- | --- | --- | --- | --- | --- | --- | --- | --- | --- | --- | --- |
| Boys | | | | | | | | | | | | | | | | | |
| 45 | 6.515 | 2.555 | -1.468 | 1.859 | 1.52 | 1.8 | 1.95 | 2.17 | 2.31 | 2.5 | 2.78 | 2.96 | 3.02 | 3.05 | 3.09 | 3.11 | 3.14 |
| 50 | 0.008 | 3.645 | -2.162 | 1.258 | 2.87 | 2.97 | 3.02 | 3.12 | 3.2 | 3.33 | 3.64 | 3.98 | 4.14 | 4.24 | 4.38 | 4.47 | 4.62 |
| 55 | 1.093 | 4.797 | -2.117 | 0.893 | 3.49 | 3.72 | 3.84 | 4.03 | 4.17 | 4.38 | 4.79 | 5.2 | 5.41 | 5.54 | 5.73 | 5.84 | 6.06 |
| 60 | 0.456 | 6.103 | -2.344 | 0.872 | 4.87 | 5.07 | 5.18 | 5.36 | 5.49 | 5.69 | 6.1 | 6.52 | 6.74 | 6.89 | 7.1 | 7.23 | 7.48 |
| 65 | 0.552 | 7.235 | -2.414 | 0.925 | 5.87 | 6.09 | 6.21 | 6.41 | 6.55 | 6.77 | 7.23 | 7.7 | 7.94 | 8.1 | 8.32 | 8.46 | 8.72 |
| 70 | 0.394 | 8.756 | -2.406 | 1.039 | 7.15 | 7.39 | 7.53 | 7.75 | 7.92 | 8.18 | 8.75 | 9.35 | 9.64 | 9.83 | 10.09 | 10.25 | 10.55 |
| 75 | 0.57 | 9.864 | -2.544 | 1.174 | 8.3 | 8.52 | 8.64 | 8.86 | 9.02 | 9.28 | 9.86 | 10.46 | 10.73 | 10.91 | 11.14 | 11.29 | 11.54 |
| 80 | 0.405 | 11.044 | -2.52 | 1.231 | 9.29 | 9.53 | 9.66 | 9.9 | 10.08 | 10.37 | 11.04 | 11.73 | 12.06 | 12.26 | 12.53 | 12.69 | 12.97 |
| 85 | 1.511 | 12.059 | -2.591 | 1.233 | 10.1 | 10.39 | 10.55 | 10.82 | 11.02 | 11.35 | 12.05 | 12.74 | 13.04 | 13.23 | 13.47 | 13.61 | 13.86 |
| 90 | 2.044 | 12.763 | -2.558 | 1.374 | 10.57 | 10.88 | 11.06 | 11.36 | 11.58 | 11.96 | 12.76 | 13.51 | 13.83 | 14.01 | 14.25 | 14.39 | 14.61 |
| 95 | -2.847 | 13.709 | -2.572 | 1.647 | 12.11 | 12.26 | 12.35 | 12.52 | 12.67 | 12.94 | 13.7 | 14.67 | 15.17 | 15.48 | 15.9 | 16.15 | 16.6 |
| Girls | | | | | | | | | | | | | | | | | |
| 45 | 2.575 | 2.625 | -2.197 | 1.729 | 1.92 | 2.02 | 2.07 | 2.17 | 2.24 | 2.36 | 2.62 | 2.84 | 2.93 | 2.98 | 3.03 | 3.06 | 3.11 |
| 50 | 1.086 | 3.572 | -2.223 | 1.309 | 2.77 | 2.88 | 2.94 | 3.05 | 3.13 | 3.27 | 3.57 | 3.87 | 4 | 4.08 | 4.18 | 4.25 | 4.35 |
| 55 | 0.666 | 4.797 | -2.24 | 1.076 | 3.74 | 3.9 | 3.99 | 4.14 | 4.24 | 4.42 | 4.79 | 5.18 | 5.36 | 5.48 | 5.64 | 5.74 | 5.92 |
| 60 | 0.311 | 5.898 | -2.321 | 0.99 | 4.72 | 4.9 | 5 | 5.17 | 5.29 | 5.48 | 5.89 | 6.33 | 6.54 | 6.69 | 6.89 | 7.01 | 7.25 |
| 65 | -0.093 | 7.062 | -2.329 | 0.911 | 5.7 | 5.91 | 6.03 | 6.22 | 6.36 | 6.58 | 7.06 | 7.57 | 7.84 | 8.02 | 8.28 | 8.45 | 8.77 |
| 70 | 0.567 | 8.585 | -2.348 | 1.096 | 6.91 | 7.16 | 7.3 | 7.53 | 7.7 | 7.98 | 8.58 | 9.2 | 9.5 | 9.69 | 9.95 | 10.11 | 10.4 |
| 75 | 0.525 | 9.616 | -2.423 | 1.021 | 7.85 | 8.12 | 8.27 | 8.52 | 8.7 | 8.99 | 9.61 | 10.25 | 10.56 | 10.76 | 11.04 | 11.22 | 11.54 |
| 80 | 0.336 | 10.775 | -2.41 | 1.1 | 8.84 | 9.12 | 9.28 | 9.54 | 9.74 | 10.06 | 10.77 | 11.51 | 11.87 | 12.1 | 12.41 | 12.61 | 12.96 |
| 85 | 0.386 | 11.791 | -2.41 | 1.085 | 9.66 | 9.97 | 10.15 | 10.44 | 10.66 | 11.01 | 11.79 | 12.59 | 12.98 | 13.23 | 13.58 | 13.79 | 14.18 |
| 90 | 1.708 | 12.683 | -2.344 | -0.002 | 8.91 | 10.07 | 10.57 | 11.24 | 11.61 | 12.07 | 12.68 | 13.26 | 13.69 | 14.01 | 14.56 | 14.96 | 15.78 |

Table S6. Standard deviation (SD) for weight-for-length in 0–24-month-old Jordanian toddlers

| Length | -3 SD | -2 SD | -1 SD | 1 SD | 2 SD | 3 SD |
| --- | --- | --- | --- | --- | --- | --- |
| Weight-for-length (boys) | | | | | | |
| 45 | 1.68 | 2.15 | 2.56 | 2.96 | 3.00 | 3.01 |
| 50 | 2.76 | 2.95 | 3.21 | 4.13 | 4.52 | 4.83 |
| 55 | 3.17 | 3.66 | 4.19 | 5.39 | 5.91 | 6.37 |
| 60 | 4.58 | 5.02 | 5.52 | 6.72 | 7.31 | 7.87 |
| 65 | 5.56 | 6.03 | 6.58 | 7.92 | 8.54 | 9.10 |
| 70 | 6.83 | 7.33 | 7.95 | 9.62 | 10.34 | 10.97 |
| 75 | 8.00 | 8.46 | 9.05 | 10.71 | 11.36 | 11.90 |
| 80 | 9.00 | 9.47 | 10.11 | 12.03 | 12.77 | 13.36 |
| 85 | 9.73 | 10.32 | 11.07 | 13.02 | 13.68 | 14.18 |
| 90 | 10.13 | 10.75 | 11.57 | 13.82 | 14.47 | 14.91 |
| 95 | 13.79 | 13.79 | 13.79 | 13.79 | 13.79 | 13.79 |
| weight-for-length (girls) | | | | | | |
| 50 | 2.63 | 2.85 | 3.14 | 3.99 | 4.27 | 4.48 |
| 55 | 3.53 | 3.86 | 4.27 | 5.35 | 5.80 | 6.19 |
| 60 | 4.48 | 4.86 | 5.31 | 6.53 | 7.09 | 7.60 |
| 65 | 5.45 | 5.86 | 6.38 | 7.83 | 8.53 | 9.21 |
| 70 | 6.55 | 7.08 | 7.73 | 9.46 | 10.20 | 10.86 |
| 75 | 7.51 | 8.05 | 8.72 | 10.54 | 11.30 | 11.95 |
| 80 | 8.46 | 9.04 | 9.77 | 11.83 | 12.70 | 13.46 |
| 85 | 9.20 | 9.86 | 10.69 | 12.92 | 13.89 | 14.73 |
| 90 | 9.27 | 10.25 | 11.32 | 13.72 | 14.71 | 15.59 |
| 95 | 9.07 | 10.60 | 11.99 | 14.45 | 15.54 | 16.56 |
| 50 | 2.63 | 2.85 | 3.14 | 3.99 | 4.27 | 4.48 |
| 55 | 3.53 | 3.86 | 4.27 | 5.35 | 5.80 | 6.19 |
| 60 | 4.48 | 4.86 | 5.31 | 6.53 | 7.09 | 7.60 |
| 65 | 5.45 | 5.86 | 6.38 | 7.83 | 8.53 | 9.21 |
| 70 | 6.55 | 7.08 | 7.73 | 9.46 | 10.20 | 10.86 |
| 75 | 7.51 | 8.05 | 8.72 | 10.54 | 11.30 | 11.95 |
| 80 | 8.46 | 9.04 | 9.77 | 11.83 | 12.70 | 13.46 |
| 85 | 9.20 | 9.86 | 10.69 | 12.92 | 13.89 | 14.73 |
| 90 | 9.27 | 10.25 | 11.32 | 13.72 | 14.71 | 15.59 |
| 95 | 9.07 | 10.60 | 11.99 | 14.45 | 15.54 | 16.56 |

S7. Length-for-age and weight-for-age up to 2 years relative to WHO growth standards

| Age | Jordanian references | | | | | WHO standards | | | | |
| --- | --- | --- | --- | --- | --- | --- | --- | --- | --- | --- |
|  | C3 | C10 | C50 | C90 | C97 | C3 | C10 | C50 | C90 | C97 |
| Length-for-age (boys) | | | | | | | | | | |
| 0 | 45.3 | 46.3 | 49 | 51.5 | 52.4 | 46.3 | 47.5 | 49.9 | 52.3 | 53.4 |
| 30 | 49.3 | 50 | 52.7 | 56 | 57.1 | 51.1 | 52.2 | 54.7 | 57.2 | 58.4 |
| 60 | 53.2 | 54.1 | 57.2 | 60.3 | 61.2 | 54.7 | 55.9 | 58.4 | 61 | 62.2 |
| 90 | 56.3 | 57.3 | 60.4 | 63.4 | 64.4 | 57.6 | 58.8 | 61.4 | 64 | 65.3 |
| 120 | 58.9 | 59.9 | 62.9 | 66.1 | 67.2 | 60 | 61.2 | 63.9 | 66.6 | 67.8 |
| 150 | 60.6 | 61.8 | 65.1 | 68.4 | 69.5 | 61.9 | 63.2 | 65.9 | 68.6 | 69.9 |
| 180 | 62.3 | 63.5 | 66.9 | 70.1 | 71.2 | 63.6 | 64.9 | 67.6 | 70.4 | 71.6 |
| 210 | 64 | 65.1 | 68.5 | 71.8 | 72.9 | 65.1 | 66.4 | 69.2 | 71.9 | 73.2 |
| 240 | 65.6 | 66.7 | 70 | 73.4 | 74.5 | 66.5 | 67.8 | 70.6 | 73.4 | 74.7 |
| 270 | 67 | 68.1 | 71.5 | 74.9 | 76.1 | 67.7 | 69.1 | 72 | 74.8 | 76.2 |
| 300 | 68.2 | 69.4 | 72.9 | 76.4 | 77.5 | 69 | 70.4 | 73.3 | 76.2 | 77.6 |
| 330 | 69.4 | 70.6 | 74.2 | 77.7 | 78.9 | 70.2 | 71.6 | 74.5 | 77.5 | 78.9 |
| 360 | 70.4 | 71.7 | 75.4 | 79 | 80.1 | 71.3 | 72.7 | 75.7 | 78.8 | 80.2 |
| 390 | 71.5 | 72.8 | 76.5 | 80.2 | 81.4 | 72.4 | 73.8 | 76.9 | 80 | 81.5 |
| 420 | 72.6 | 73.9 | 77.6 | 81.3 | 82.5 | 73.4 | 74.9 | 78 | 81.2 | 82.7 |
| 450 | 73.6 | 74.9 | 78.7 | 82.4 | 83.7 | 74.4 | 75.9 | 79.1 | 82.4 | 83.9 |
| 480 | 74.6 | 75.9 | 79.6 | 83.4 | 84.7 | 75.4 | 76.9 | 80.2 | 83.5 | 85.1 |
| 510 | 75.5 | 76.8 | 80.6 | 84.4 | 85.8 | 76.3 | 77.9 | 81.2 | 84.6 | 86.2 |
| 540 | 76.3 | 77.6 | 81.5 | 85.4 | 86.8 | 77.2 | 78.8 | 82.3 | 85.7 | 87.3 |
| 570 | 77.1 | 78.4 | 82.3 | 86.4 | 87.8 | 78.1 | 79.7 | 83.2 | 86.8 | 88.4 |
| 600 | 77.8 | 79.1 | 83.1 | 87.4 | 88.8 | 78.9 | 80.6 | 84.2 | 87.8 | 89.5 |
| 630 | 78.4 | 79.7 | 83.9 | 88.3 | 89.8 | 79.7 | 81.5 | 85.1 | 88.8 | 90.5 |
| 660 | 78.9 | 80.2 | 84.6 | 89.2 | 90.8 | 80.5 | 82.3 | 86 | 89.8 | 91.6 |
| 690 | 79.4 | 80.7 | 85.2 | 90.1 | 91.7 | 81.3 | 83.1 | 86.9 | 90.8 | 92.6 |
| 720 | 79.8 | 81.1 | 85.8 | 91 | 92.7 | 82.1 | 83.9 | 87.8 | 91.7 | 93.6 |
| Weight-for-age (boys) | | | | | | | | | | |
| 0 | 2.3 | 2.5 | 3 | 3.7 | 3.9 | 2.5 | 2.8 | 3.3 | 4 | 4.3 |
| 30 | 3.4 | 3.6 | 4.3 | 5 | 5.3 | 3.4 | 3.8 | 4.5 | 5.3 | 5.7 |
| 60 | 4.3 | 4.6 | 5.4 | 6.2 | 6.5 | 4.4 | 4.7 | 5.6 | 6.5 | 7 |
| 90 | 5.1 | 5.4 | 6.2 | 7.1 | 7.4 | 5.1 | 5.5 | 6.4 | 7.4 | 7.9 |
| 120 | 5.6 | 6 | 6.9 | 7.8 | 8.1 | 5.6 | 6 | 7 | 8.1 | 8.6 |
| 150 | 6.1 | 6.4 | 7.4 | 8.4 | 8.8 | 6.1 | 6.5 | 7.5 | 8.6 | 9.2 |
| 180 | 6.4 | 6.8 | 7.8 | 8.9 | 9.4 | 6.4 | 6.9 | 7.9 | 9.1 | 9.7 |
| 210 | 6.7 | 7.1 | 8.2 | 9.4 | 9.8 | 6.7 | 7.2 | 8.3 | 9.5 | 10.2 |
| 240 | 7.1 | 7.5 | 8.7 | 9.9 | 10.3 | 7 | 7.5 | 8.6 | 9.9 | 10.5 |
| 270 | 7.5 | 7.9 | 9.1 | 10.3 | 10.8 | 7.2 | 7.7 | 8.9 | 10.2 | 10.9 |
| 300 | 7.8 | 8.3 | 9.5 | 10.7 | 11.2 | 7.5 | 8 | 9.2 | 10.5 | 11.2 |
| 330 | 8.1 | 8.5 | 9.8 | 11 | 11.5 | 7.7 | 8.2 | 9.4 | 10.8 | 11.5 |
| 360 | 8.3 | 8.8 | 10 | 11.3 | 11.8 | 7.8 | 8.4 | 9.6 | 11.1 | 11.8 |
| 390 | 8.6 | 9 | 10.3 | 11.6 | 12.1 | 8 | 8.6 | 9.9 | 11.4 | 12.1 |
| 420 | 8.8 | 9.2 | 10.5 | 11.9 | 12.5 | 8.2 | 8.8 | 10.1 | 11.6 | 12.4 |
| 450 | 9 | 9.4 | 10.8 | 12.2 | 12.8 | 8.4 | 9 | 10.3 | 11.9 | 12.7 |
| 480 | 9.2 | 9.6 | 11 | 12.5 | 13 | 8.5 | 9.1 | 10.5 | 12.1 | 12.9 |
| 510 | 9.4 | 9.9 | 11.2 | 12.7 | 13.3 | 8.7 | 9.3 | 10.7 | 12.4 | 13.2 |
| 540 | 9.6 | 10.1 | 11.4 | 12.9 | 13.5 | 8.9 | 9.5 | 10.9 | 12.6 | 13.5 |
| 570 | 9.8 | 10.2 | 11.6 | 13.1 | 13.7 | 9 | 9.7 | 11.1 | 12.9 | 13.7 |
| 600 | 9.9 | 10.4 | 11.8 | 13.3 | 13.8 | 9.2 | 9.8 | 11.3 | 13.1 | 14 |
| 630 | 10 | 10.5 | 11.9 | 13.4 | 14 | 9.3 | 10 | 11.5 | 13.3 | 14.3 |
| 660 | 10.1 | 10.6 | 12.1 | 13.6 | 14.2 | 9.5 | 10.2 | 11.8 | 13.6 | 14.5 |
| 690 | 10.1 | 10.6 | 12.2 | 13.8 | 14.5 | 9.7 | 10.3 | 12 | 13.8 | 14.8 |
| 720 | 10.1 | 10.7 | 12.3 | 14 | 14.7 | 9.8 | 10.5 | 12.2 | 14.1 | 15.1 |
| Length-for-age (girls) | | | | | | | | | | |
| 0 | 46.1 | 46.8 | 49 | 51.1 | 51.7 | 45.6 | 46.8 | 49.1 | 51.5 | 52.7 |
| 30 | 48.3 | 49.1 | 51.9 | 55.1 | 56.2 | 50 | 51.2 | 53.7 | 56.2 | 57.4 |
| 60 | 52.1 | 53.1 | 56.2 | 59.3 | 60.2 | 53.2 | 54.5 | 57.1 | 59.7 | 60.9 |
| 90 | 54.8 | 55.9 | 59 | 61.9 | 62.9 | 55.8 | 57.1 | 59.8 | 62.5 | 63.8 |
| 120 | 57.4 | 58.4 | 61.5 | 64.5 | 65.6 | 58 | 59.3 | 62.1 | 64.9 | 66.2 |
| 150 | 59.3 | 60.4 | 63.5 | 66.7 | 67.7 | 59.9 | 61.2 | 64 | 66.9 | 68.2 |
| 180 | 60.7 | 61.8 | 65.1 | 68.3 | 69.3 | 61.5 | 62.8 | 65.7 | 68.6 | 70 |
| 210 | 62.1 | 63.2 | 66.7 | 69.9 | 70.9 | 62.9 | 64.3 | 67.3 | 70.3 | 71.6 |
| 240 | 63.6 | 64.8 | 68.3 | 71.6 | 72.6 | 64.3 | 65.7 | 68.7 | 71.8 | 73.2 |
| 270 | 65.2 | 66.4 | 69.9 | 73.3 | 74.4 | 65.6 | 67 | 70.1 | 73.2 | 74.7 |
| 300 | 66.6 | 67.8 | 71.4 | 74.9 | 76.1 | 66.8 | 68.3 | 71.5 | 74.6 | 76.1 |
| 330 | 67.9 | 69.2 | 72.8 | 76.4 | 77.6 | 68 | 69.5 | 72.8 | 76 | 77.5 |
| 360 | 69 | 70.3 | 74 | 77.7 | 78.9 | 69.2 | 70.7 | 74 | 77.3 | 78.9 |
| 390 | 70 | 71.3 | 75.1 | 78.8 | 80.1 | 70.3 | 71.8 | 75.2 | 78.6 | 80.2 |
| 420 | 70.9 | 72.3 | 76.2 | 79.9 | 81.2 | 71.3 | 72.9 | 76.4 | 79.8 | 81.4 |
| 450 | 71.9 | 73.3 | 77.2 | 81 | 82.2 | 72.4 | 74 | 77.5 | 81 | 82.7 |
| 480 | 72.8 | 74.3 | 78.2 | 82 | 83.3 | 73.3 | 75 | 78.6 | 82.2 | 83.9 |
| 510 | 73.8 | 75.3 | 79.2 | 83 | 84.3 | 74.3 | 76 | 79.7 | 83.3 | 85 |
| 540 | 74.9 | 76.3 | 80.2 | 84.1 | 85.5 | 75.2 | 77 | 80.7 | 84.4 | 86.2 |
| 570 | 75.9 | 77.2 | 81.2 | 85.2 | 86.6 | 76.2 | 77.9 | 81.7 | 85.5 | 87.3 |
| 600 | 76.7 | 78 | 82 | 86.2 | 87.7 | 77 | 78.8 | 82.7 | 86.6 | 88.4 |
| 630 | 77.4 | 78.7 | 82.8 | 87.1 | 88.6 | 77.9 | 79.7 | 83.7 | 87.6 | 89.4 |
| 660 | 77.8 | 79.2 | 83.5 | 87.9 | 89.5 | 78.7 | 80.6 | 84.6 | 88.6 | 90.5 |
| 690 | 78 | 79.5 | 84 | 88.5 | 90.1 | 79.6 | 81.5 | 85.5 | 89.6 | 91.5 |
| 720 | 77.8 | 79.5 | 84.4 | 89 | 90.6 | 80.3 | 82.3 | 86.4 | 90.6 | 92.5 |
| Weight-for-age (girls) | | | | | | | | | | |
| 0 | 2.1 | 2.2 | 2.9 | 3.8 | 3.9 | 2.4 | 2.7 | 3.2 | 3.9 | 4.2 |
| 30 | 3.1 | 3.3 | 4 | 4.6 | 4.9 | 3.2 | 3.5 | 4.2 | 5 | 5.4 |
| 60 | 3.9 | 4.2 | 4.9 | 5.6 | 5.8 | 4 | 4.3 | 5.1 | 6 | 6.5 |
| 90 | 4.7 | 4.9 | 5.7 | 6.5 | 6.8 | 4.6 | 5 | 5.8 | 6.9 | 7.4 |
| 120 | 5.2 | 5.5 | 6.3 | 7.2 | 7.5 | 5.1 | 5.5 | 6.4 | 7.5 | 8.1 |
| 150 | 5.6 | 5.9 | 6.8 | 7.7 | 8.1 | 5.5 | 5.9 | 6.9 | 8.1 | 8.7 |
| 180 | 5.9 | 6.3 | 7.2 | 8.3 | 8.7 | 5.8 | 6.2 | 7.3 | 8.5 | 9.2 |
| 210 | 6.2 | 6.6 | 7.6 | 8.7 | 9.2 | 6.1 | 6.5 | 7.6 | 8.9 | 9.6 |
| 240 | 6.6 | 6.9 | 8 | 9.2 | 9.7 | 6.3 | 6.8 | 7.9 | 9.3 | 10 |
| 270 | 6.9 | 7.3 | 8.4 | 9.7 | 10.1 | 6.6 | 7 | 8.2 | 9.6 | 10.4 |
| 300 | 7.3 | 7.7 | 8.8 | 10.1 | 10.5 | 6.8 | 7.3 | 8.5 | 9.9 | 10.7 |
| 330 | 7.5 | 7.9 | 9.1 | 10.4 | 10.8 | 7 | 7.5 | 8.7 | 10.2 | 11 |
| 360 | 7.7 | 8.2 | 9.4 | 10.7 | 11.1 | 7.1 | 7.7 | 8.9 | 10.5 | 11.3 |
| 390 | 8 | 8.4 | 9.7 | 11 | 11.5 | 7.3 | 7.9 | 9.2 | 10.8 | 11.6 |
| 420 | 8.2 | 8.6 | 9.9 | 11.2 | 11.8 | 7.5 | 8 | 9.4 | 11 | 11.9 |
| 450 | 8.4 | 8.8 | 10.1 | 11.5 | 12 | 7.7 | 8.2 | 9.6 | 11.3 | 12.2 |
| 480 | 8.6 | 9 | 10.4 | 11.8 | 12.3 | 7.8 | 8.4 | 9.8 | 11.5 | 12.5 |
| 510 | 8.8 | 9.3 | 10.6 | 12.1 | 12.6 | 8 | 8.6 | 10 | 11.8 | 12.7 |
| 540 | 9 | 9.5 | 10.8 | 12.3 | 12.8 | 8.2 | 8.8 | 10.2 | 12 | 13 |
| 570 | 9.2 | 9.6 | 11 | 12.5 | 13 | 8.3 | 8.9 | 10.4 | 12.3 | 13.3 |
| 600 | 9.3 | 9.8 | 11.2 | 12.7 | 13.3 | 8.5 | 9.1 | 10.6 | 12.5 | 13.5 |
| 630 | 9.5 | 10 | 11.4 | 12.9 | 13.5 | 8.7 | 9.3 | 10.9 | 12.8 | 13.8 |
| 660 | 9.6 | 10.1 | 11.5 | 13.1 | 13.6 | 8.8 | 9.5 | 11.1 | 13 | 14.1 |
| 690 | 9.7 | 10.2 | 11.6 | 13.2 | 13.8 | 9 | 9.7 | 11.3 | 13.3 | 14.3 |
| 720 | 9.8 | 10.3 | 11.8 | 13.3 | 13.9 | 9.2 | 9.8 | 11.5 | 13.5 | 14.6 |

Table S8. Weight-for-length of children from birth up to 2 years relative to WHO growth standards

| Length | Jordanian references | | | | | WHO standards | | | | |
| --- | --- | --- | --- | --- | --- | --- | --- | --- | --- | --- |
|  | C3 | C10 | C50 | C90 | C97 | C3 | C10 | C50 | C90 | C97 |
| Weight-for-length (boys) | | | | | | | | | | |
| 45.0 | 1.8 | 2.2 | 2.8 | 3.1 | 3.1 | 2.1 | 2.2 | 2.4 | 2.8 | 2.9 |
| 50.0 | 3.0 | 3.1 | 3.6 | 4.2 | 4.5 | 2.8 | 3.0 | 3.3 | 3.7 | 4.0 |
| 55.0 | 3.7 | 4.0 | 4.8 | 5.5 | 5.8 | 3.9 | 4.1 | 4.5 | 5.1 | 5.4 |
| 60.0 | 5.1 | 5.4 | 6.1 | 6.9 | 7.2 | 5.1 | 5.4 | 6.0 | 6.7 | 7.0 |
| 65.0 | 6.1 | 6.4 | 7.2 | 8.1 | 8.5 | 6.3 | 6.6 | 7.3 | 8.1 | 8.5 |
| 70.0 | 7.4 | 7.8 | 8.8 | 9.8 | 10.3 | 7.2 | 7.6 | 8.4 | 9.4 | 9.9 |
| 75.0 | 8.5 | 8.9 | 9.9 | 10.9 | 11.3 | 8.2 | 8.6 | 9.5 | 10.6 | 11.2 |
| 80.0 | 9.5 | 9.9 | 11.0 | 12.3 | 12.7 | 9.0 | 9.4 | 10.4 | 11.6 | 12.3 |
| 85.0 | 10.4 | 10.8 | 12.1 | 13.2 | 13.6 | 9.9 | 10.4 | 11.5 | 12.8 | 13.5 |
| 90.0 | 10.9 | 11.4 | 12.8 | 14.0 | 14.4 | 11.0 | 11.5 | 12.7 | 14.1 | 14.9 |
| 95.0 | 12.3 | 12.5 | 13.7 | 15.5 | 16.2 | 12.0 | 12.6 | 13.9 | 15.4 | 16.2 |
| Weight-for-length (girls) | | | | | | | | | | |
| 45.0 | 2.10 | 2.25 | 2.72 | 3.09 | 3.18 | 2.10 | 2.20 | 2.50 | 2.80 | 2.90 |
| 50.0 | 2.88 | 3.05 | 3.57 | 4.08 | 4.24 | 2.80 | 3.00 | 3.40 | 3.80 | 4.00 |
| 55.0 | 3.90 | 4.14 | 4.80 | 5.48 | 5.75 | 3.90 | 4.10 | 4.50 | 5.10 | 5.40 |
| 60.0 | 4.91 | 5.17 | 5.90 | 6.69 | 7.02 | 5.00 | 5.20 | 5.90 | 6.60 | 7.00 |
| 65.0 | 5.92 | 6.22 | 7.06 | 8.03 | 8.45 | 6.00 | 6.30 | 7.10 | 8.00 | 8.50 |
| 70.0 | 7.15 | 7.53 | 8.58 | 9.68 | 10.12 | 6.90 | 7.30 | 8.20 | 9.20 | 9.70 |
| 75.0 | 8.13 | 8.52 | 9.61 | 10.77 | 11.21 | 7.80 | 8.20 | 9.10 | 10.30 | 10.90 |
| 80.0 | 9.11 | 9.54 | 10.76 | 12.09 | 12.61 | 8.60 | 9.00 | 10.10 | 11.30 | 12.00 |
| 85.0 | 9.95 | 10.43 | 11.77 | 13.21 | 13.78 | 9.50 | 10.00 | 11.20 | 12.60 | 13.30 |
| 90.0 | 10.38 | 11.01 | 12.54 | 14.02 | 14.60 | 10.60 | 11.20 | 12.50 | 14.00 | 14.80 |

Table S9. Summary Statistics of Quantile Residuals for Growth Models by Sex and Indicator

| Growth Indicator | Sex | Mean Residual | Variance | Skewness | Kurtosis | Filliben Correlation |
| --- | --- | --- | --- | --- | --- | --- |
| Height-for-Age | Boys | 0.0014 | 0.9915 | –0.0016 | 2.9032 | 0.9986 |
| Weight-for-Age |  | –0.0009 | 0.9958 | –0.0013 | 2.8791 | 0.9989 |
| Weight-for-Height |  | –0.0008 | 0.9968 | –0.0004 | 2.9061 | 0.9994 |
| Height-for-Age | Girls | –0.0005 | 0.9918 | –0.00009 | 2.8876 | 0.9985 |
| Weight-for-Age |  | –0.0007 | 0.9956 | –0.0014 | 2.8823 | 0.9989 |
| Weight-for-Height |  | –0.0010 | 0.9966 | –0.0007 | 2.8965 | 0.9993 |

Table S10. Sample distribution across centiles for each model

| Centile (%) | Boys | | | Girls | | |
| --- | --- | --- | --- | --- | --- | --- |
|  | Height-for-Age | Weight-for-Age | Weight-for-Length | Height-for-Age | Weight-for-Age | Weight-for-Length |
| 3 | 3.69 | 3.56 | 2.99 | 3.71 | 3.38 | 3.38 |
| 5 | 5.80 | 5.62 | 5.36 | 5.85 | 5.53 | 5.65 |
| 10 | 10.48 | 10.18 | 10.73 | 10.28 | 10.39 | 10.25 |
| 25 | 23.83 | 24.00 | 27.19 | 23.45 | 23.93 | 23.55 |
| 50 | 50.19 | 50.00 | 49.47 | 49.71 | 50.51 | 49.80 |
| 75 | 76.42 | 76.08 | 74.25 | 76.14 | 75.68 | 75.97 |
| 90 | 89.75 | 89.42 | 88.43 | 89.90 | 89.53 | 89.16 |
| 95 | 94.36 | 94.52 | 94.16 | 94.36 | 94.29 | 94.04 |
| 97 | 96.42 | 96.48 | 97.36 | 96.31 | 96.36 | 96.82 |
